# Kinin receptors regulate skeletal muscle regeneration: differential effects for B1 and B2 receptors

**DOI:** 10.1007/s00011-023-01766-4

**Published:** 2023-07-18

**Authors:** Leonardo Martins, Weslley Wallace Amorim, Marcos Fernandes Gregnani, Ronaldo de Carvalho Araújo, Fatimunnisa Qadri, Michael Bader, João Bosco Pesquero

**Affiliations:** 1 Division of Medical Sciences, Laboratory of Transcriptional Regulation, Institute of Medical Biology of Polish Academy of Sciences (IMB-PAN), 3a Tylna St., 90-364 Łódź, Poland; 2 Center for Research and Molecular Diagnosis of Genetic Diseases, Federal University of São Paulo, Rua Pedro de Toledo 669, 9th Floor, São Paulo, 04039032 Brazil; 3 Department of Biochemistry and Molecular Biology, Federal University of São Paulo, Rua Três de Maio 100, 4th Floor, São Paulo, 04044-020 Brazil; 4 Laboratory of Exercise Genetics and Metabolism, Federal University of São Paulo, Rua Pedro de Toledo 669, 9th Floor, São Paulo, 04039032 Brazil; 5 Max-Delbrück Center for Molecular Medicine (MDC), Robert-Rössle-Str. 10, 13125 Berlin, Germany; 6 Institute for Biology, University of Lübeck, Ratzeburger Allee 160, 23562 Lübeck, Germany; 7 Charité University Medicine Berlin, Charitéplatz 1, 10117 Berlin, Germany; 8 German Center for Cardiovascular Research (DZHK), Potsdamer Str. 58, 10785 Berlin, Germany; 9 Department of Biophysics, Federal University of São Paulo, Rua Botucatu 862, 6th Floor, São Paulo, 04023-062 Brazil

**Keywords:** Fibrosis, Skeletal muscle, Injury, Myogenesis, Kinin, Contusion

## Abstract

**Objective and design:**

After traumatic skeletal muscle injury, muscle healing is often incomplete and produces extensive fibrosis. Bradykinin (BK) reduces fibrosis in renal and cardiac damage models through the B2 receptor. The B1 receptor expression is induced by damage, and blocking of the kallikrein-kinin system seems to affect the progression of muscular dystrophy. We hypothesized that both kinin B1 and B2 receptors could play a differential role after traumatic muscle injury, and the lack of the B1 receptor could produce more cellular and molecular substrates for myogenesis and fewer substrates for fibrosis, leading to better muscle healing.

**Material and methods:**

To test this hypothesis, tibialis anterior muscles of kinin receptor knockout animals were subjected to traumatic injury. Myogenesis, angiogenesis, fibrosis, and muscle functioning were evaluated.

**Results:**

Injured B1KO mice showed a faster healing progression of the injured area with a larger amount of central nucleated fiber post-injury when compared to control mice. In addition, they exhibited higher neovasculogenic capacity, maintaining optimal tissue perfusion for the post-injury phase; had higher amounts of myogenic markers with less inflammatory infiltrate and tissue destruction. This was followed by higher amounts of SMAD7 and lower amounts of p-SMAD2/3, which resulted in less fibrosis. In contrast, B2KO and B1B2KO mice showed more severe tissue destruction and excessive fibrosis. B1KO animals had better results in post-injury functional tests compared to control animals.

**Conclusions:**

We demonstrate that injured skeletal muscle tissues have a better repair capacity with less fibrosis in the presence of B2 receptor and absence of B1 receptor, including better performances in functional tests.

**Supplementary Information:**

The online version contains supplementary material available at 10.1007/s00011-023-01766-4.

## Introduction

At all stages of life tissues as bones, muscles, tendons, cartilage, and ligaments are vulnerable to injury and disease. Between 60 and 77% of injuries involve the musculoskeletal system. Muscle injuries often require medical care and are difficult to treat, resulting in both absences from and disabilities at work, leading to a significant public health burden [1]. Specifically, in sports medicine, muscle injury is the most frequent event among athletes of various modalities, accounting for 55% of all cases and occurs in both recreational and competitive activities [2]. Recent studies involving professional soccer athletes revealed that in over 500,000 h of play there may be more than 4500 injuries, 35.2% muscle injuries and an average of two injuries per season for each player. This situation leads to 27% of activity removals due to muscle injuries mainly in lower limbs and due to re-injury [3, 4]. The healing process of the injury, especially those of greater severity, is slow. The intense fibrosis, associated with atrophy and muscle weakness, impairs the recovery of functionality and facilitates the occurrence of re-injury [5]. The picture of muscle injury is complex and involves connective tissue, blood vessels and neural components, in addition to myocytes. The tissue repair process involves a set of steps from muscle injury, such as: muscle degeneration with inflammation and edema; muscular regeneration with fibrosis and partial or total recovery of the injured muscles. In most of these processes the boundaries are not very well defined, with overlap of steps occurring [6]. Muscle injury consists of the destruction of the integrity of the sarcolemma and of the basal lamina, which triggers the necrosis of myofibers. Local edema and hematoma allow local proteases to act promoting autodigestion, which is the main process of muscle degeneration. These processes attract inflammatory cells, mainly neutrophils, in the early hours, which bring large amounts of proteases, vasodilators like NO (nitric oxide), and angiogenic factors like VEGF (vascular endothelial growth factor) [7–9].

Several studies have focused on the role of the renin-angiotensin system (RAS) and the process of fibrosis [10–12]. The pro-fibrotic function of the RAS is opposed by another vasoactive peptide system, the kallikrein-kinin system (KKS). The main effector of the KKS is bradykinin (BK), a nonapeptide produced from kininogen by plasma and tissue kallikreins. Angiotensin-converting enzyme (ACE, also known as kininase-II) can degrade BK with high efficiency [13, 14]. Kinins act through two G protein-coupled receptors: the B2 receptor (BDKRB2 or B2R) and the B1 receptor (BDKRB1 or B1R), which are both expressed in most tissues, including skeletal muscle [13–16]; and present increased expression in response to damage and inflammatory processes [17, 18]. Different studies suggest that BK has a role as an antifibrotic agent. In vivo studies in hepatic, renal and cardiac fibrosis models show that the infusion of BK reduces fibrosis and TGF-β levels [19–21]. On the other hand, the blockade of tissue kallikrein increases damage and fibrosis in kidney [22]. B2R knockout mice (B2KO) show increased cardiac and renal fibrosis [23–25], and the overexpression of tissue kallikrein reduces the fibrotic phenotype and TGF-β levels in renal and cardiac fibrosis models [19, 26, 27]. Since the KKS has anti-fibrotic effects in other tissues, its components are present in skeletal muscle [15], and the pharmacological blockade of the BK receptors causes increased damage and fibrosis and reduction of dystrophic skeletal muscle strength [16], we hypothesized that the KKS may also play a role in post-injury musculoskeletal repair. Therefore, we submitted knockout mice for each and both kinin-receptors to muscular injury and evaluated how the lack of these receptors affects the healing process. We found that each of the receptors can affect the tissue repair process in different ways at the site of injury over time. This differential action of kinin receptors on skeletal muscle repair should be considered in the development of new therapies directed to the skeletal muscle system.

## Results

### Lack of kinin receptors increases damage and fibrosis of injured muscle

To evaluate whether the KKS plays a role in the progression of muscle injury, muscle of kinin receptor knockout mice were subjected to traumatic injury, as shown in the schematic diagram (Fig. S1). The injured area was red and swollen soon after impact, but no sign of bone fracture was observed during manual examination. The swelling lasted approximately four days, and the reddened area completely disappeared on the 7th day. Deletion of B1 and B2 kinin receptors in the mice was confirmed by PCR genotyping, as demonstrated in Fig. S1.

**Fig. 1:**
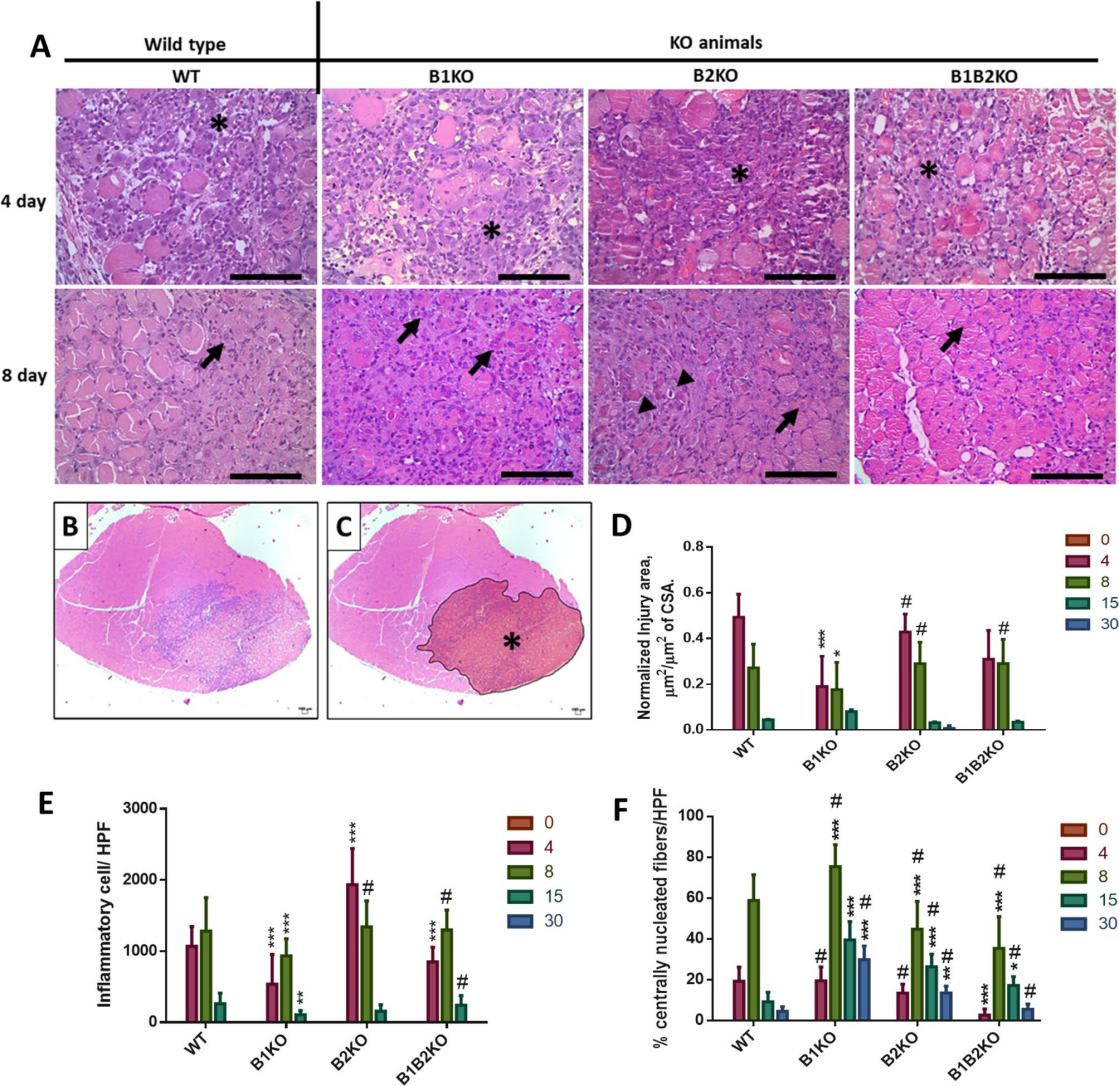
Lack of kinin B2 but not B1 receptors increases damage of injured muscle. **A** Representative photos from H&E staining of Tibial anterior (TA) muscles for all animal groups at indicated days after injury showing an inflammatory area (*), fibers centrally nucleated (arrow) and necrotic cells (arrowhead) and **B** and **C** injured area delimited (*). (Scale bars = 100 μm). (*n* = 5, all groups). **D** TA injury area normalized to cross-sectional area (square micrometer per square micrometer). Data are represented as mean ± SD. *P < 0.05 KO groups vs WT control group. #P < 0.05 B1KO vs other KOs. **E** Number of inflammatory cells within the areas of injury. High-power fields (HPF). Data are represented as mean ± SD. *P < 0.05 KO groups vs WT control group. #P < 0.05 B1KO vs others KOs. **F** Percentage of centronucleated cells within the areas of injury. High-power fields (HPF). Data are represented as mean ± SD. *P < 0.05 KO groups vs WT control group. # P < 0.05 all KO groups vs KO groups

We observed that B2KO mice showed increased tissue damage in the injured skeletal muscle as assessed by macroscopic evaluation and H&E staining (Fig. S2 and 1A). There was an increase of mononucleated cells that infiltrated the muscle, and also an evident increase of necrotic-degenerating cells in comparison with the control wild type (WT) mice (Fig. 1A).

Histomorphometry of the injury areas (Fig. 1A, B and C) revealed that B1KO animals showed smaller injury area compared to WT (at day 4 and 8 post-injury), while B2KO and B1B2KO animals showed equivalent areas (0.19 ± 0.13 and 0.17 ± 0.11 (B1KO) vs; 0.42 ± 0.07 and 0.29 ± 0.09 (B2KO); 0.31 ± 0.12 and 0.29 ± 0.10 (B1B2KO), μm^2^/μm^2^ CSA; respectively) (Fig. 1D). The dynamics of inflammatory infiltrates in areas of injury were also different between KO groups compared to WT, this followed the expected dimensional profile for an acute inflammatory event, both in histomorphometric and visual analyses. B1KO animals showed less inflammatory cells in areas of injury compared to WT on days 4, 8 and 15 post-injury (536.7; 933.5 and 105.6 vs 1068.1; 1281.1 and 261.3 inflammatory cells/HPF, respectively), while the B2KO group showed an increase on days 4 and 8 post-injury (1932.8 and 1342 inflammatory cells/HPF, respectively). B1B2KO group showed less inflammation only on day 4 compared to WT with an increase on day 8 and 15 compared to B1KO (848.9 ± 203.9 vs WT; 1297.8 ± 281.5 and 240.4 ± 135.4 vs B1KO) (Fig. 1E). Regarding the number of centronucleated fibers (CNFs) found in areas of injury, the B1KO group showed a higher number compared to the WT, B2KO, and B1B2KO groups, especially at days 4, 8, 15 and 30 post-injury (19.4 ± 6.7; 75.3 ± 10.7; 39.5 ± 8.8 and 29.8 ± 6.7 (B1KO) vs 19.2 ± 6.9; 58.7 ± 12.6; 9.2 ± 4.6 and 4.5 ± 2.2 (WT); 13.4 ± 4.3; 44.7 ± 13.6; 26.3 ± 6.1 and 13.5 ± 3.4 (B2KO); 2.74 ± 2.9; 35.3 ± 15.4; 17.2 ± 4.3; 5.57 ± 2.5 (B1B2KO), % CNFs) (Fig. 1F).

The analysis of collagen deposition in the perimysial and endomysial zones are important to determine the fibrotic potential in the post-injury scar repair, because they are areas adjacent to the muscle fibers and their clusters. In general, collagen deposition in the areas of injury were abundant and homogeneous from day 4 to 8, characteristic of fibroplasia, followed by a reorganization and accumulation within the scar zones on days 15 and 30 in all groups (Fig. S3A). We performed collagen differentiation under polarized light of the collagen deposition zones. At first, the images corroborate what was observed in light photomicrographs, however, a lower refringence is observed in the tissues of B2KO animals for days 15 and 30 and in B1B2KO animals on day 30. It is also noted that the spreading and diffusion of fibers on days 4 and 8 post-injury, are reorganized and compacted around the muscle structures on days 15 and 30 (Fig. S3B).

**Fig. 2:**
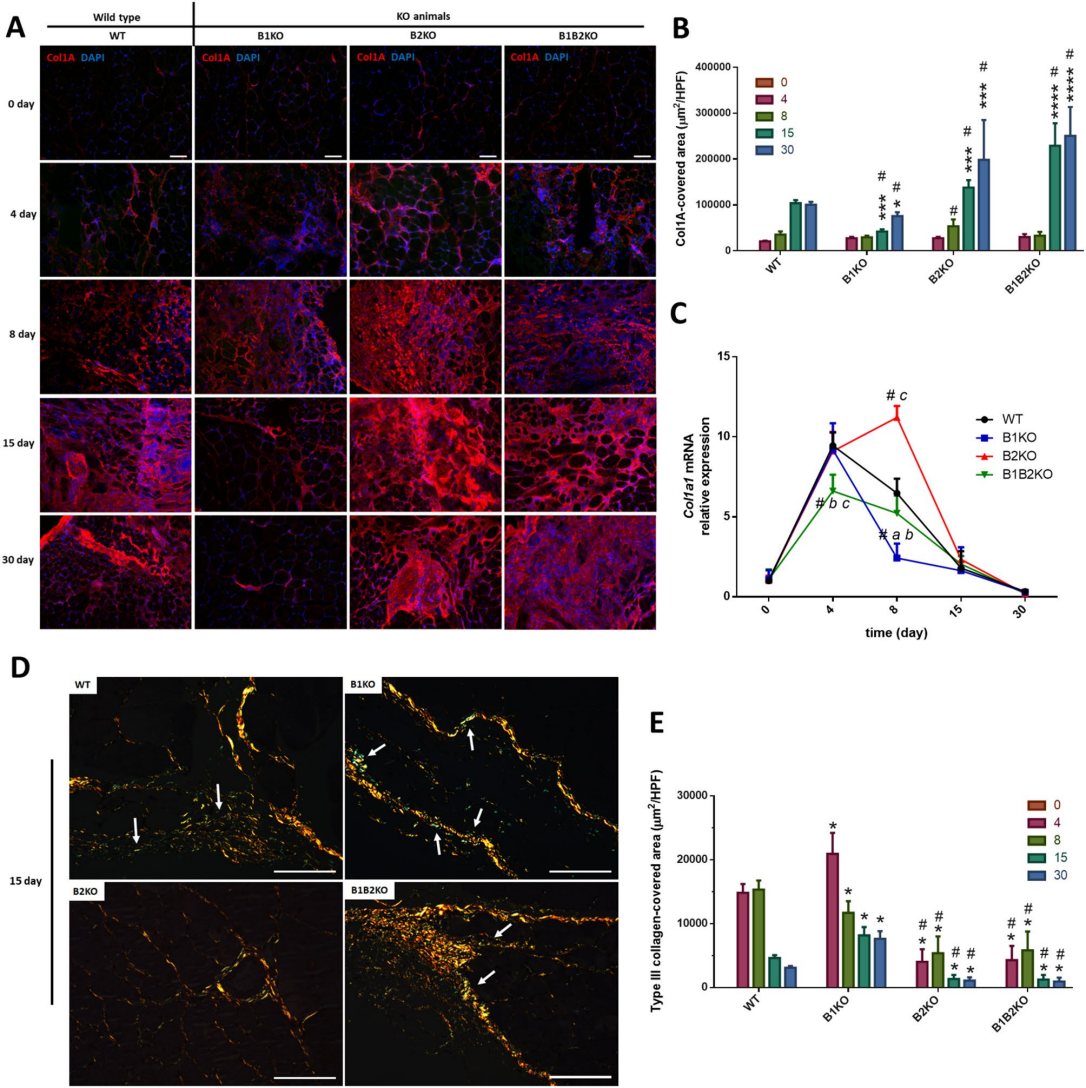
Lack of kinin B2 receptors but not B1 receptor increases fibrosis of injured muscle. **A** Representative immunofluorescence analyses of Col1A in the injured skeletal muscle at indicated days after injury and non-injured tissue (0 day) in each group (*n* = *5,* for all groups). Col1A (red), DAPI, 4’,6-diamidino-2-phenylindole stain (blue). (Scale bars = 100 μm.) **B** Collagen deposition area. High-power fields (HPF). Data are represented as mean ± SD. *P < 0.05 KO groups vs WT control group. #P < 0.05 B1KO vs other KOs. **C** Relative expression of *Col1a1* mRNA was detected at lower levels in B1KO group and high levels in B2KO group at 8th day after injury (*n* = 5 mice per group) compared with each other (#, P < 0.05.) and only in the B1KO group compared to WT control (*, P < 0.05). **D** Representative Sirius red staining combined with polarized light microscopy for collagen fiber structure. Polarized images of the Picrosirius Red stained sections were quantified to evaluate collagen differentiation by fiber length, width, and angle. Yellow–red strong birefringence (type I collagen) and greenish color with thinner fibers (type III collagen) (white arrow) (Scale bars = 100 μm.) **E** Type III collagen area by High-power fields (HPF). Data are represented as mean ± SD. *P < 0.05. KO groups vs WT control group. #P < 0.05 all KO groups vs KO groups (color figure online)

The same pattern found in histomorphometry from light microscopy with H&E, was confirmed by immunolabeling for COL1A in areas of injury (Fig. 2A). All injury borders have been identified and delimited (Fig. S3C). The B2KO group showed a progressive increase in collagen deposition from day 8 post-injury, being significantly higher in relation to the B1KO animals from day 8 (1.8-fold-higher), and in relation to the WT group from day 15 (1.3-fold-higher). The B1B2KO group showed higher collagen deposition from day 15 compared to the WT and B1KO groups (2.2-fold-higher and 5.4-fold-higher, respectively). Interestingly, the B1KO group showed a significant decrease in collagen deposition compared to WT animals and other KOs from day 15 (2.4-fold-lower (WT); 5.4-fold-lower (B1B2KO); 3.2-fold-lower (B2KO)) (Fig. 2B). The results of the expression of *Col1a1* revealed that all groups increased the expression of this gene on day 4 post-injury, as expected for the pattern of injury obtained, however, on day 8 post-injury the B2KO group showed a significant increase in relation to the WT group, while the B1KO group showed a significant decrease (1.7-fold-higher and 2.7-fold-lower, respectively) (Fig. 2C). The expression data corroborates the Col1A pattern obtained by immunolabeling. Interestingly, on collagen differentiation from slides stained with Sirius Red and polarized light, especially at day 15 post-injury, the B1KO group showed higher amounts of type III collagen in lesion areas (Fig. 2D and E).

### Lack of *Bdkrb2* increases TGF-β1 levels and phospho-Smad2/3 dependent signaling while *Bdkrb1* increases Smad7 in injured muscle

**Fig. 3:**
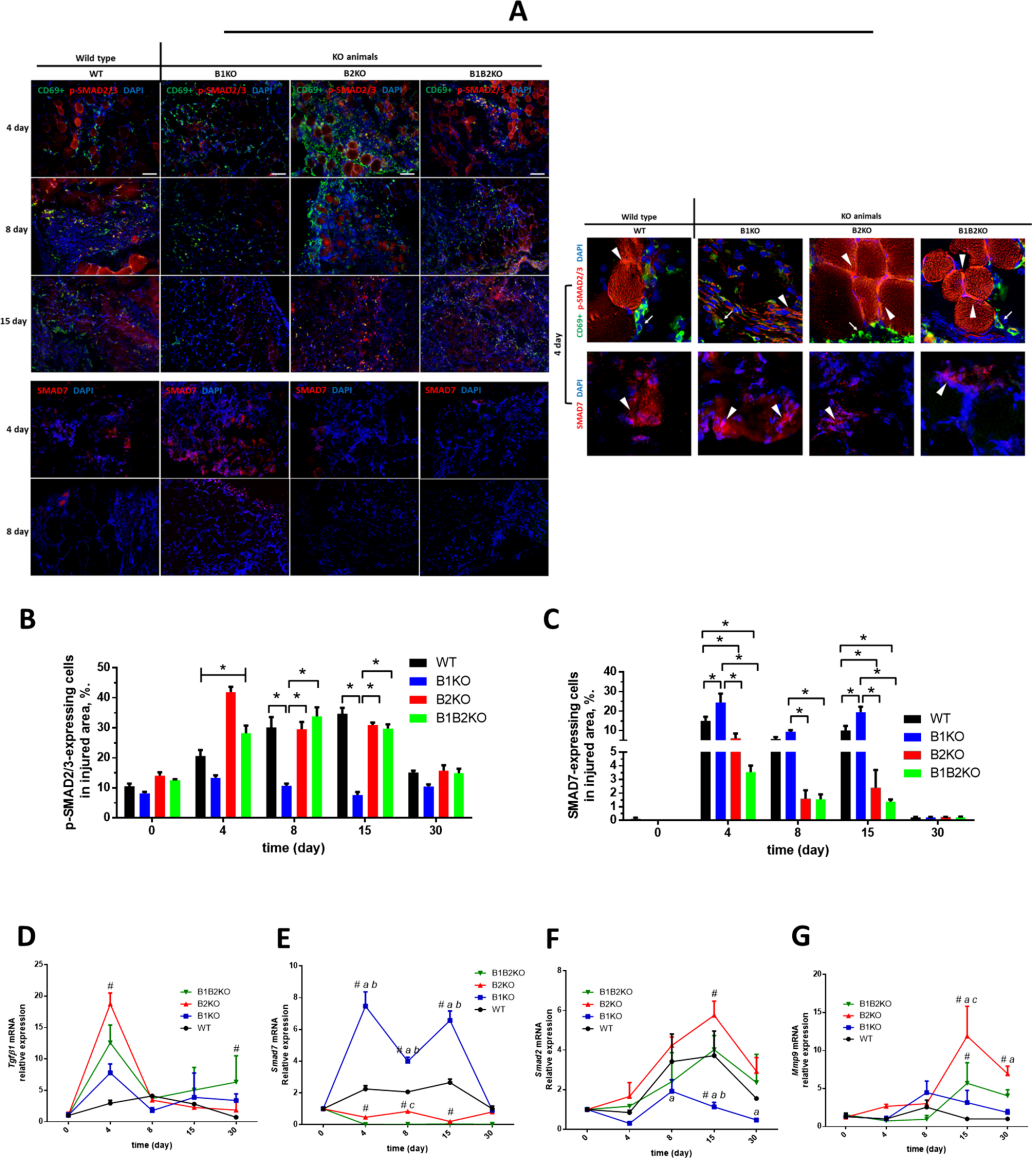
Lack of B2 enhances the activation of the Tgf-β/Smad pathway, whereas the lack of B1 stimulates the production of inhibitory Smad (SMAD7). **A** Representative immunofluorescence analyses of CD69 + cells (green, arrow), p-SMAD2/3 (red, arrowhead) (Top), and SMAD7 (red, arrowhead) (Bottom) in the injured skeletal muscle at indicated days after injury in each group (*n* = *5* per group). CD69 (green), p-SMAD2/3 (red, arrowhead, top), and SMAD7 (red, arrowhead, bottom). DAPI, 4’,6-diamidino-2-phenylindole stain (blue). (Scale bars = 100 μm.) **B** p-SMAD2/3 quantification in the injured skeletal muscle after injury. Data are expressed as percentage of p-SMAD2/3-expressing cells in each group (mean ± SE) (*, P < 0.05). **C** SMAD7 quantification in the injured skeletal muscle at 4, 8 and 15 days after injury. Data are expressed as percentage of Smad7-expressing cells in each group (mean ± SE) (*, P < 0.05). No positive markings were found on days 0 and 30. **D**, **E**, **F** and **G** Relative expressions of *Tgfβ1*, *Smad7*, *Smad2* and *Mmp9* mRNA were quantified by RT-qPCR. Values (mean ± SD) normalized to 18 s rRNA, (*n* = 5 mice per group) compared KO group vs WT (*P < 0.05, **P < 0.01, ***P < 0.001) or each other (#, P < 0.05) (color figure online)

To analyze the histomorphometric characteristics of post-injury tissue fibrosis, we determined the amount of p-SMAD2/3 and SMAD7 (Fig. 3A) by immunolabeling, as well as the gene expression of some genes associated with the fibrogenic process (Fig. 3D–G). Compared with the control group, a significant increase of p-SMAD2/3 in the B2KO and B1B2KO groups was observed on day 4 post-injury (41.9 ± 7.7 (B2KO) and 28.2 ± 11.4 (B1B2KO) vs 20.6 ± 8.8 (WT)), while in the B1KO group there was a decrease (13.3 ± 3.4 (B1KO) vs 20.6 ± 8.8 (WT)). At day 8 and 15 post-injury, the B1KO group maintained low p-SMAD2/3 levels compared to the WT group, while the B2KO and B1B2KO animals showed no difference from the control group (10.7 ± 3.1 and 7.5 ± 4.5 (B1KO) vs 30.1 ± 15.2 and 34.4 ± 8.8 (WT); 29.5 ± 10.9 and 30.9 ± 3.5 (B2KO) and 33.8 ± 13.3 and 29.8 ± 6.1 (B1B2KO) vs 30.1 ± 15.2 and 34.4 ± 8.8 (WT); for day 8 and 15 post-injury, respectively) (Fig. 3B).

In immunolabeling for SMAD7, only the B1KO group showed a significant increase by about 10% at day 4 and 15 post-injury when compared to the WT control group (Fig. 3C). The B2KO and B1B2KO groups showed a significant decrease in the amount of Smad7 present in areas of injury compared to the WT control group and the B1KO group on days 4 and 15 post-injury (23.5 ± 14.2 and 18.5 ± 8.7 (B1KO;) 14.1 ± 6.9 and 9.8 ± 7.4 (WT); 6.1 ± 7.8 and 2.5 ± 4.1 (B2KO) and 3.3 ± 1.5 and 1.2 ± 0.5 (B1B2KO), % of cells expressing Smad7 in areas of injury for day 4 and 8, respectively), and only relative to or B1KO group on day 8 (Fig. 3C).

Regarding *Tgfβ1* expression in post-injury muscle tissue, all KO animals for kinin receptors showed an increase on day 4, however, with proportional differences. The B2KO group showed sixfold higher *Tgfβ1* expression at day 4 post-injury compared to the WT group (18.74 ± 1.74 vs 2.94 ± 0.46, respectively). Concomitantly, this group showed decreased *Smad7* expression at all times post-injury, with increased *Smad2* expression over time at an average of 2.75-fold higher compared to the WT group. Conversely to what was observed in the B2KO group, B1KO animals had the highest *Smad7* mRNA expression values from day 4 to day 15 (*vs* other groups) at an average of 2.5-fold higher compared to the WT group, with lower *Smad2* mRNA expression at day 15 and 30 post-injury at an average of 3.3-fold lower than the WT group and about 5.0 and 6.2-fold lower than the B2KO group at the same experimental times (Fig. 3D–F). B2KO and B1B2KO animals showed 11.8-fold and 5.6-fold higher *Mmp9* mRNA expression than WT group at day 15, respectively (Fig. 3E).

### Lack of kinin receptors increases inflammatory cells in injured muscle

**Fig. 4:**
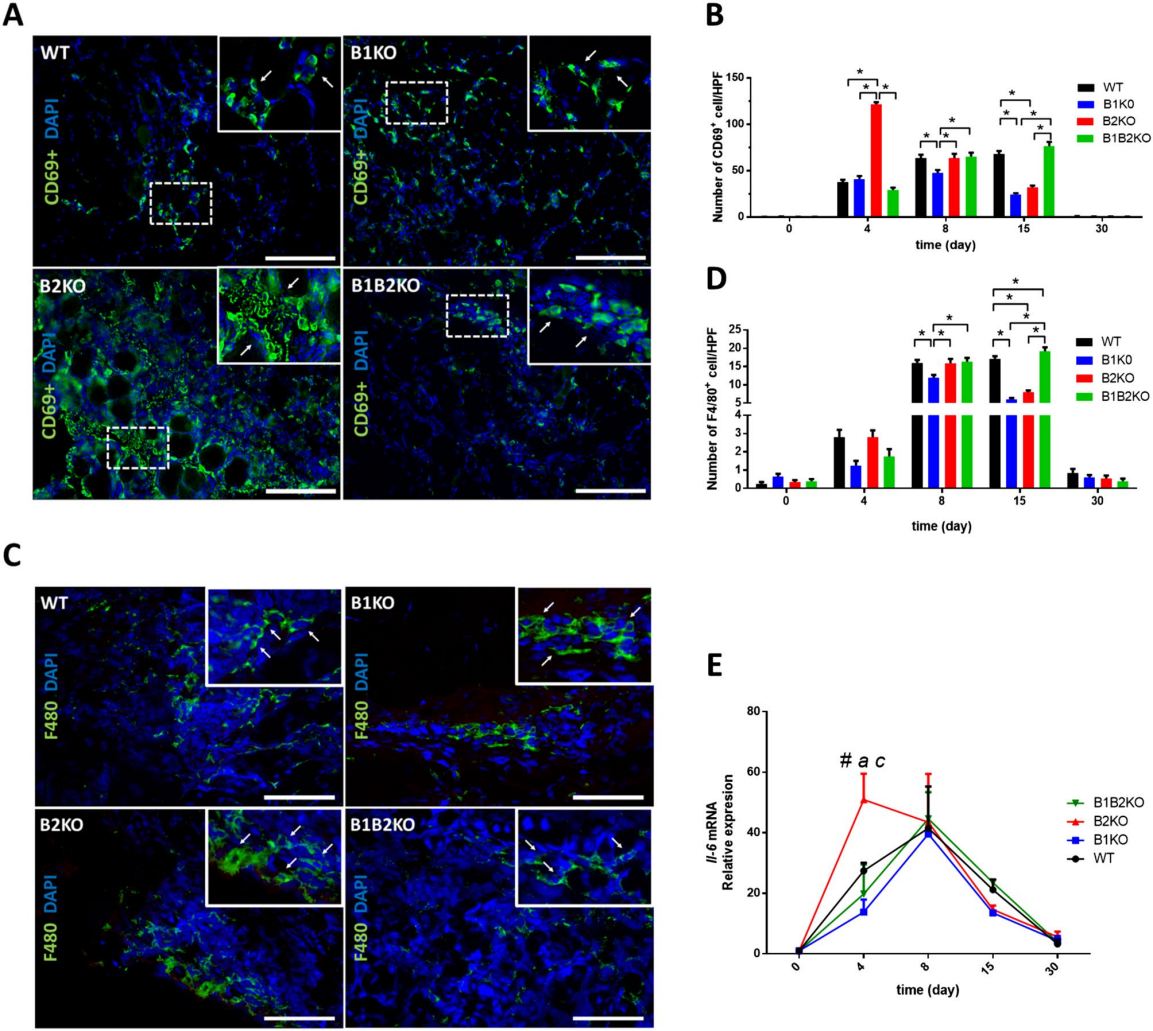
**A** Representative immunofluorescence analyses of CD69 + cells (green, white arrows) in the injured skeletal muscle at indicated days after injury (*n* = *5* per group). DAPI, 4’,6-diamidino-2-phenylindole stain (blue). (Scale bars = 100 μm.) **B** Number of CD69 + cells in injured skeletal muscle. Data are expressed by the number of CD69-expressing cells in each group per High-power fields (HPF) (mean ± SE) (*, P < 0.05). **C** Representative immunofluorescence analyses of F4/80 (green, white arrows) in the injured skeletal muscle at indicated days after injury (*n* = *5* per group). DAPI, 4’,6-diamidino-2-phenylindole stain (blue). (Scale bars = 100 μm.) **D** Number of F4/80 + cells in injured skeletal muscle. Data are expressed by the number of F4/80-expressing cells in each group per High-power fields (HPF) (mean ± SE) (*, P < 0.05). **E** Relative expression of *Il-6* mRNA was increased on the 4th day after the injury only in the B2KO group, compared to the other groups. Relative expression of *Il-6* mRNA was quantified by RT-qPCR. Values (mean ± SD) normalized to 18 s rRNA (*n* = 5 mice per group) (*, P < 0.05) (color figure online)

We performed CD69 immunolabeling in the injured skeletal muscle tissue (Fig. 4A) and observed that the number of CD69 + cells in the injured skeletal muscle of B2KO animals is increased about 3.4-fold at day 4 post-injury compared to the average of the other groups (121.6 ± 10 vs 36.1 ± 12) and decreased on 15 day when compared to WT animals (Fig. 4B). B1KO group showed no differences at day 4 post-injury compared to WT, however, at the two consecutive times of day 8 and day 15 post-injury, it showed a reduction in the number of CD69 + cells on the order of 1.3-fold and 2.7-fold, respectively, compared to WT (47.8 ± 13 and 24.4 ± 6 *vs* 53.7 ± 15 and 68.2 ± 13, respectively) (Fig. 4B).

F4/80 is a well-characterized and extensively referenced murine macrophage marker [28–30] and we determined the number of F4/80 + cells in areas of injury (Fig. 4D). All groups had a low number of F4/80 + cells on day 4 post-injury, followed by an increase on day 8, as expected. No significant difference was observed on day 4, however, on day 8 we observed a decrease in the population of these cells in the B1KO group. Such a decrease progressed on day 8 with about 64% fewer cells being compared with the WT group at the same time. Interestingly, the B2KO animals also showed at day 15 post-injury a decrease of about 50% compared to WT. A similar decrease was observed with respect to CD69 + cells from day 8 to day 15 post-injury in this group, suggesting that in the absence of the B2 receptor a threshold for supporting inflammatory cells in post-injured skeletal muscle is established after week 1 (Fig. 4B). The mRNA levels for IL-6 at day 4 post-injury in the knockout groups were consistent with the WT group throughout all experimental times, except for the B2KO group at day 4, which showed relative expression values 2.7-fold higher than the average of the other groups (Fig. 4E).

### Lack of *Bdkrb1* but not *Bdkrb2* kinin receptors increases angiogenesis and MHC production in injured muscle.

**Fig. 5:**
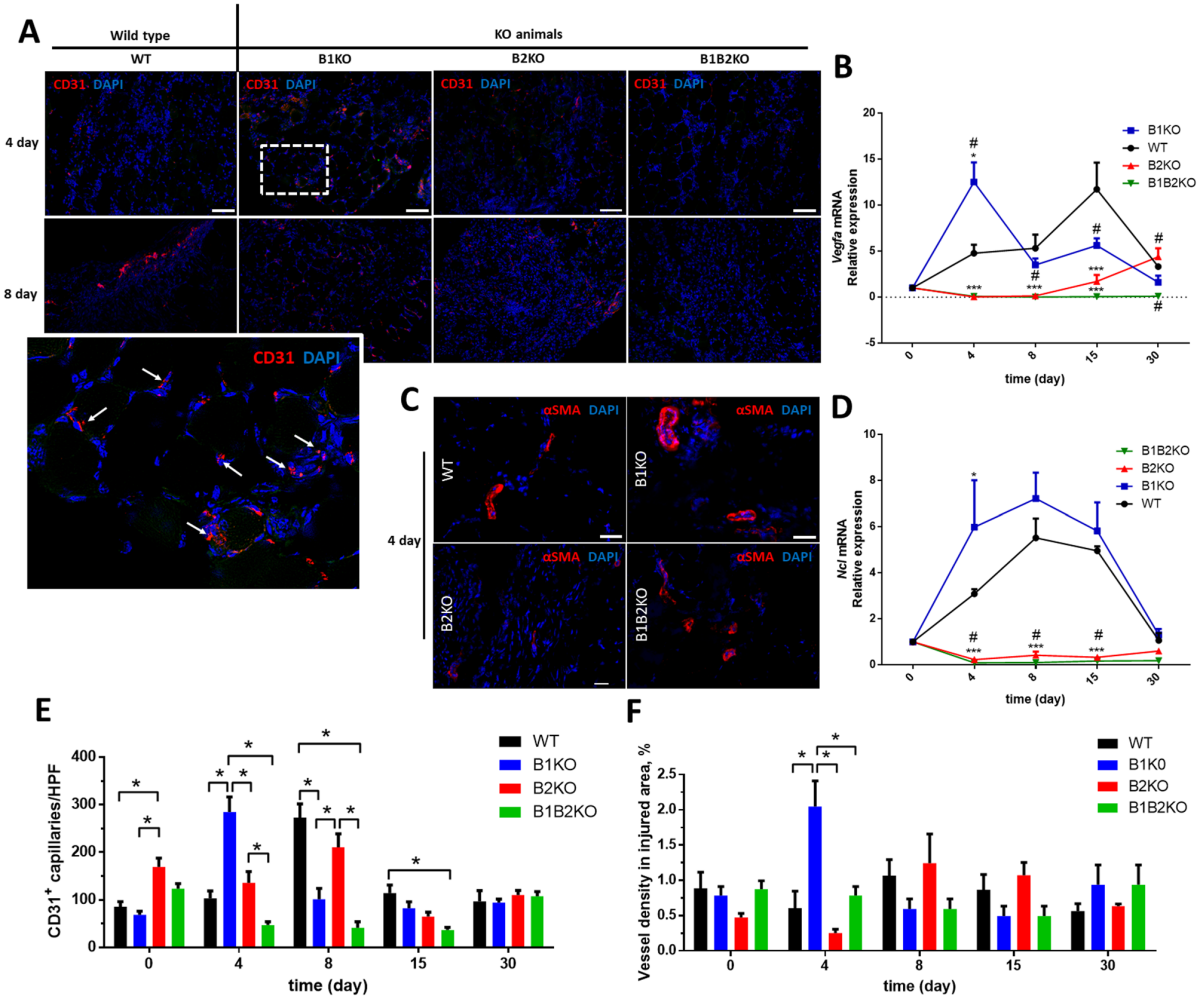
**A** Representative immunofluorescence for endothelial cells (CD31 +) (red, arrow) in the injured skeletal muscle at indicated days after injury (*n* = *5* per group). DAPI, 4’,6-diamidino-2-phenylindole stain (blue). (Scale bars = 100 μm.) **B** Relative expression of *Vegfa* mRNA was increased on the 4th day after the injury only in the B1KO group, compared to the other groups (#, P < 0.05.). Relative expression of *Vegfa* mRNA were quantified by RT-qPCR. Values (mean ± SD) normalized to 18 s rRNA (*n* = 5 mice per group) (***, P < 0.001). **C** Representative immunofluorescence for αSMA (red) in the injured skeletal muscle at indicated days after injury (*n* = *5* per group). DAPI, 4’,6-diamidino-2-phenylindole stain (blue). (Scale bars = 100 μm.) **D** Relative expression of *Ncl* mRNA was increased on the 4th day after the injury only in the B1KO group, compared to the other groups (#, P < 0.05.). Relative expression of *Ncl* mRNA was quantified by RT-qPCR. Values (mean ± SD) normalized to 18 s rRNA (*n* = 5 mice per group) (***, P < 0.001). **E** Number of CD631 + capillaries per HPF in injured skeletal muscle. Data as expressed mean ± SE (*n* = *5* per group) (*, P < 0.05). **F** αSMA-positive vessel (< 50 m outside diameter) in injured skeletal muscle. Data as expressed mean ± SE (*n* = *5* per group) (*, P < 0.05) (color figure online)

To determine capillary density, we performed immunolabeling with anti-CD31 antibody and the positively labeled capillaries in each group. CD31 + cells are normally juxtaposed to myofibers in the cross-sectional area of skeletal muscle (Fig. 5A). We observed that the B1KO group was the only group to show about a 2.7-fold increase in the number of CD31 + cells (newly formed capillaries) at day 4 post-injury compared to the WT group (Fig. 5E). This result becomes even more interesting when comparing the number of more calibrous blood vessels (vessels positively labeled for alpha-smooth muscle actin; αSMA +) (Fig. 5C) for determining vascular density in areas of injury, in which the B1KO group showed an increase in vascular density on day 4 post-injury relative to the other groups. While the average area occupied by larger vessels was around 0.5% in the other groups, the B1KO group presented 2% of its area, equivalent to 4 times more than the other groups (Fig. 5F).

B1KO animals showed twice as much *Vegfa* and *Ncl* expression on day 4 post-injury compared to WT animals (2.6-fold-higher and 1.9-fold-higher, respectively). None of the *Bdkrb2* knockout animals (B2KO and B1B2KO) showed detectable expression of these genes in areas of muscle injury, showing relative expression values ten times lower compared to WT animals (Fig. 5B, D).

**Fig. 6:**
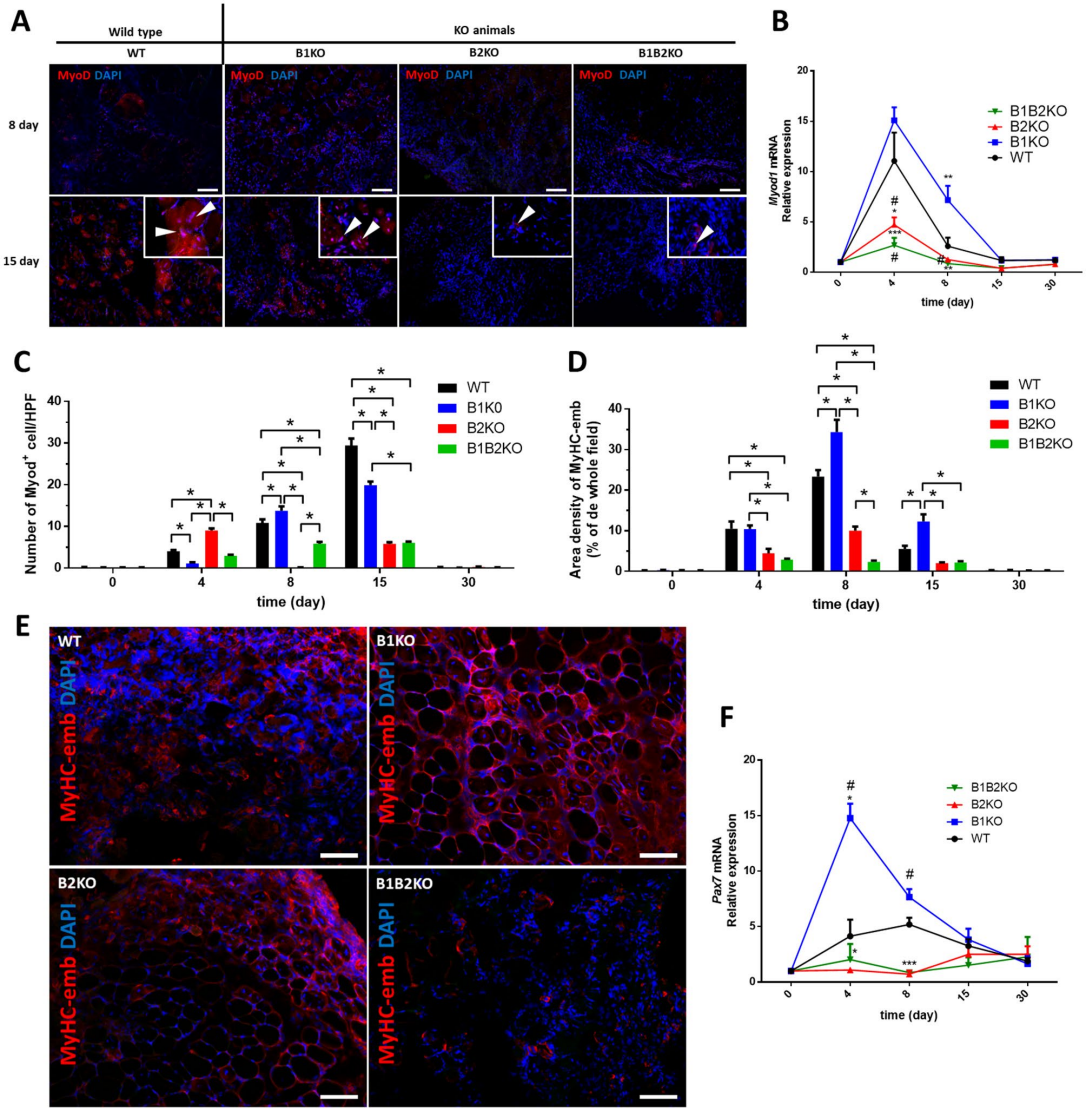
**A** Representative immunofluorescence for Myod (red, arrowhead) in the injured skeletal muscle at indicated days after injury (*n* = *5* per group). DAPI, 4’,6-diamidino-2-phenylindole stain (blue). (Scale bars = 100 μm.) **B** Relative expression of *Myod1* mRNA was increased on the 4th day after the injury in the B1KO group as observed in WT group, compared to the other groups. Relative expression of *Myod1* mRNA was quantified by RT-qPCR. Values (mean ± SD) normalized to 18 s rRNA (*n* = 5 mice per group) (*P < 0.05, **P < 0.01, ***P < 0.001) or each other (#, *P* < 0.05) **C** Number of Myod + cells per HPF in injured skeletal muscle. Data as expressed mean ± SE (*n* = *5* per group) (*, P < 0.05). **D** Density of the regeneration marker MyHC-emb in injured skeletal muscle. Data as expressed mean ± SE (*n* = *5* per group) (*, P < 0.05). **E** Immunostaining for the regeneration marker MyHC-emb (red). There was no detectable expression of MyHC-emb in uninjured TA muscle of either genotype at 0 day (supplementary). By contrast, the expression of MyHC-emb was clearly evident on the 8-day-injured TA muscle sections (*n* = *5* per group). DAPI, 4’,6-diamidino-2-phenylindole stain (blue). (Scale bars = 100 μm) **F** Relative expression of *Pax7* mRNA was increased on the 4th day after the injury in the B1KO group, compared to the other groups. Relative expression of *Pax7* mRNA was quantified by RT-qPCR. Values (mean ± SD) normalized to 18 s rRNA (*n* = 5 mice per group) (*P < 0.05, **P < 0.01, ***P < 0.001) or each other (#, *P* < 0.05) (color figure online)

To determine the myogenic potential of skeletal muscle tissues, we counted the number of MyoD + cells of each group within the lesion areas (Fig. 6A) and observed that animals from the B2KO group presented 2.2-fold more cells on the 4th post-injury day compared with WT, followed by absence of MyoD + cells on the 8th post-injury day. The opposite was observed in the B1KO groups, which showed 3.6-lower MyoD + cells on post-injury day 4, followed by 1.2-higher compared to WT on day 8. At day 15 post-injury, all KO models showed lower MyoD + cell values than the WT control group, with a notable difference in the B2KO e B1B2KO groups (1.4-fold-lower (B1KO); 5.0-fold-lower (B2KO and B1B2KO) (Fig. 6C).

Since SMA expression precedes the differentiation of skeletal myoblasts, both in vitro and after implantation in vivo [31, 32], we investigated the number of myoblasts/muscle precursor cells in injured areas by immunolabeling with α-SMA which showed a similar pattern to those obtained with MyoD immunolabeling confirming the presence of precursor cells in injured areas and the pattern reversed in the temporal analysis of these cells between B1KO and B2KO groups at day 4 and 8 post-injury. Except on day 4, B1KO showed more and B2KO and B1B2KO fewer α-SMA^+^ cells on days 8 and 15 compared to the control (Fig. S4).

Regeneration of myofibers after injury involves the hierarchical expression of the myogenic regulatory factors Myf5, MyoD, and myogenin, as well as embryonic/developmental myosin heavy chain (MyHC-emb) [33–38]. For incremental analysis of the regenerative potential of post-injury skeletal muscles, we determined the amount of MyHC-emb within the injury areas at each experimental time and in all groups (Fig. 6D, E). At day 4 post-injury, the B1KO group showed no differences compared to the WT group, however, the group increased 1.5-fold on day 8 and 2.2-fold on day 15. B2KO group showed approximately 2.3-fold lower density from day 4 to day 15, and B1B2KO did not vary in same time maintaining 2% density (Fig. 6D). These data corroborate the histomorphometric observations presented at the beginning of the study, which pointed to a higher regenerative potential in the B1KO group post-injury in the quantification of center nucleated fibers (CNFs) in lesion areas (Fig. 1F).

The expression of *Pax7* was dramatically higher in the B1KO animals compared to the other groups on day 4 (14.77 ± 1.30-fold (B1KO); 4.13 ± 1.49-fold (WT); 1.09 ± 0.12-fold (B2KO); 2.03 ± 1.41-fold (B1B2KO)). They also showed increased *Myod1* expression on day 8 compared to the other groups (7.16 ± 1.42-fold (B1KO); 2.59 ± 0.83-fold (WT); 1.26 ± 0.0-fold (B2KO); 0.86 ± 0.28-fold (B1B2KO)). Animals knocked out for the B2 receptor (B2KO and B1B2KO) showed lower relative expression than the B1KO and WT groups at all times for both genes (Fig. 6B, F).

### Lack of *Bdkrb2* reduces the performance and functionality of the injured muscle

**Fig. 7:**
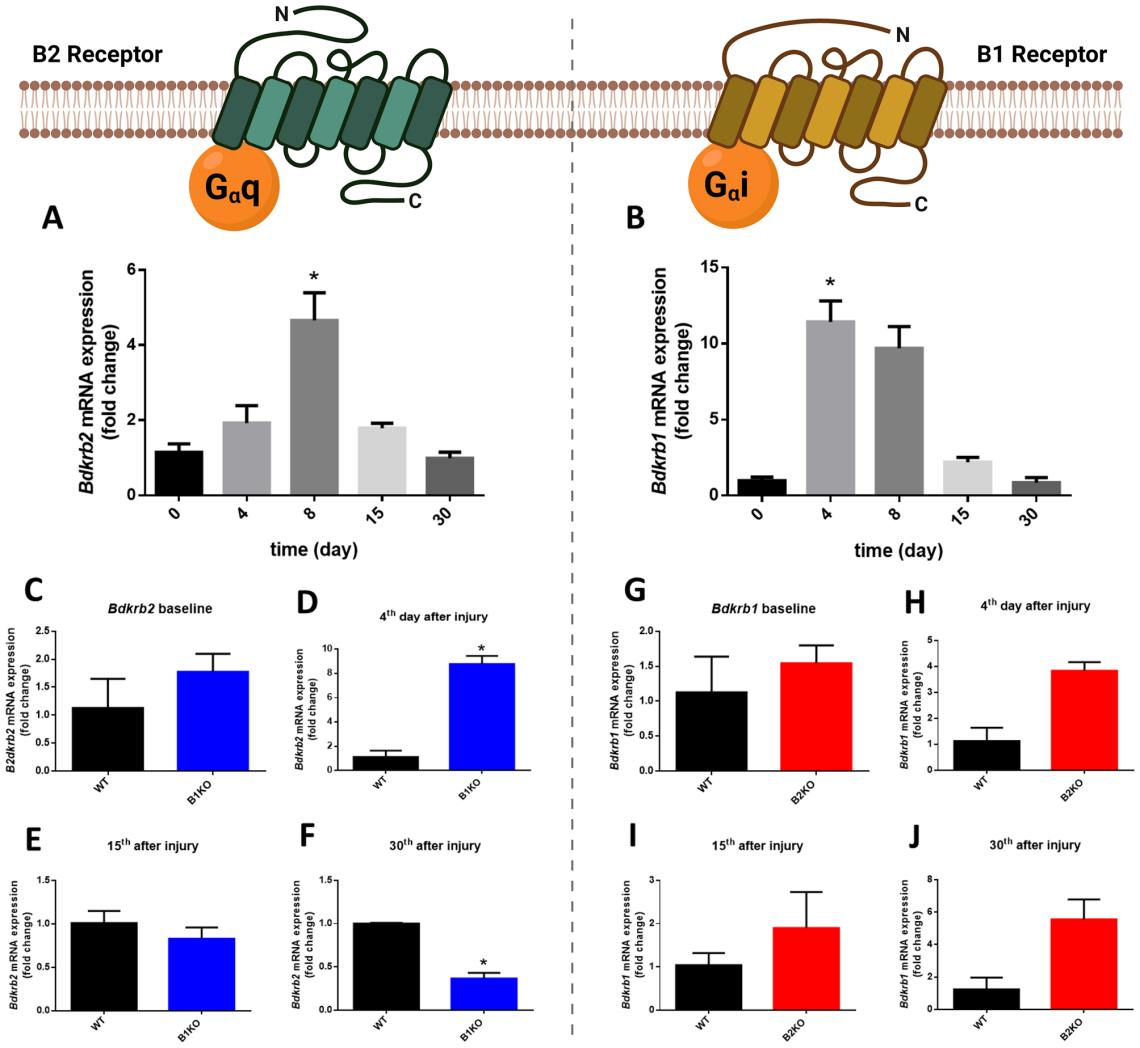
Relative expression of **A** *Bdkrb2* (B2R) and **B** *Bdkrb1* (B1R) mRNA in the injured skeletal muscle in the WT group. Relative expression of B2R mRNA for B1KO vs WT (**C**–**F**) and relative expression of B1R mRNA for B2KO vs WT (**G**–**J**). Relative expression of *Bdkrb2 and Bdkrb1* mRNA were quantified by RT-qPCR. Values (mean ± SE) normalized to 18 s rRNA (*n* = 5 mice per group) (*, P < 0.05)

We determined the *Bdkrb2* and *Bdkrb1* expression by RT-PCR over the course of the injury and the control animals increased B2 receptor expression on day 8 post-injury and B1 receptor on day 4 (4.66 ± 1.46-fold and 11.43 ± 2.76-fold, respectively) (Fig. 7A, B). B1 receptor expression in B2KO animals increased relative to the WT group between days 4 and 15 post-injury, but this augment was not statistically significant (Fig. 7G–J). On the other hand, interestingly, B1KO animals showed an increase in B2R expression on day 4 post-injury and a significant decrease relative to the WT group on day 30 post-injury (8.77 ± 0.95-fold and 0.36 ± 0.09-fold, respectively) (Fig. 7C–F).

**Fig. 8:**
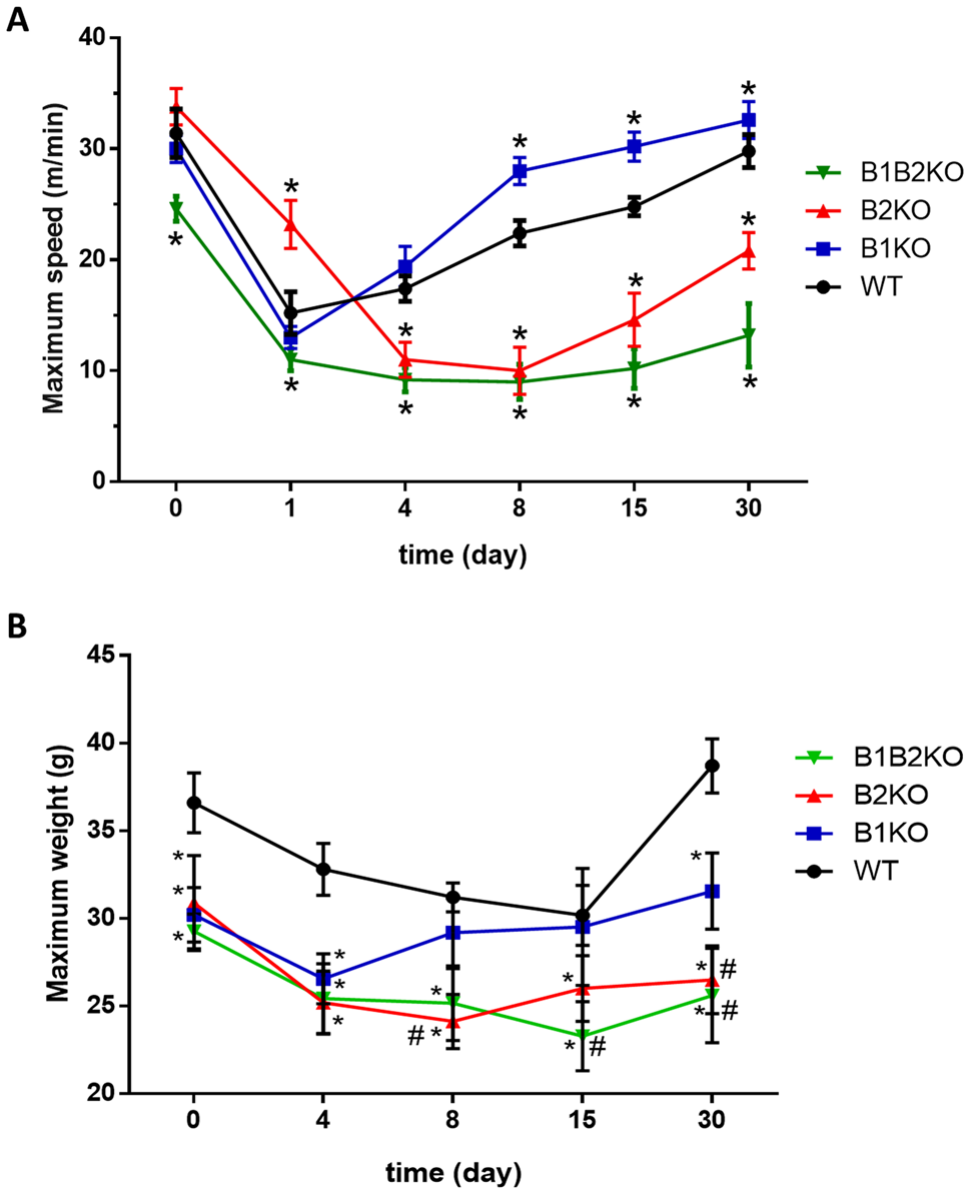
Treadmill running ability (**A**) and climbing strength/endurance test combined with incremental mass series (**B**) were tested in all groups in functional analyzes. (*n* = 10 mice per group) compared KO group vs WT (*P < 0.05) or each other (#, P < 0.05). Data presented as mean ± SD

Regarding the performance measured in physical tests such as endurance stress test 24 h post-injury and on days 4, 8, 15 and 30 post-injury, all groups showed a decrease in performance 24 h after injury, as expected. From the first 24 h to the 4th day post-injury, we observed a continuity between the values of B1KO and WT, while the B2KO animals showed a drop of about 67.5% in their performance relative to before the injury. The WT and B1KO animals recovered about 71.1% and 93.3% of their performance on day 8, respectively. The recovery of the B1KO group was 1.3-fold higher than the WT group on day 8. The B2KO and B1B2KO groups showed a prolonged decline with 46.4% and 38.5% loss of maximum speed capacity 30 days post-injury, respectively. The B1KO animals, on the other hand, from day 8 post-injury, showed 1.25-fold higher velocity when compared with WT animals, including positive yields in relation to that found before the injury on the order of 0.6% and 8.3% for days 15 and 30 post-injury (Fig. 8A). Regarding performance on a vertical ladder to gauge maximum loaded weight, interestingly, all KO animals showed lower values at all times compared to WT animals. However, the WT, B2KO and B1B2KO groups showed progressively lower values until day 8 compared to pre-injury (1.15-fold-lower; 1.28-fold-lower; 1.16-fold-lower, respectively). Additionally, the B1KO group showed similar performance on day 8 compared to pre-injury (30.20 ± 1.54 and 29.18 ± 2.04, respectively). Only B2KO and B1B2KO groups showed significantly lower performance than pre-injury at the last time point (1.16-fold-lower and 1.14-fold-lower, respectively) (Fig. 8B). There were no significant changes in any of the normalized muscle weights in all groups (Fig. S5).

## Discussion

It has been described that BK reduces fibrosis in renal and cardiac damage models through the B2 receptor [19, 21, 39], as well as B1 receptor expression is induced by damage and inflammation [13, 17, 40, 41], and that blocking of the KKS seems to have an effect on the progression of muscular dystrophy [16]. Thus, we hypothesized that BK receptors, as well as the KKS, could be playing a role in skeletal muscle repair.

Excessive evidence has shown that fibrogenesis is a broad process involving different biological systems, such as renin-angiotensin system, inflammation and oxidative stress, transforming growth factor β (TGF-β)/Smad signaling, Wnt/β-catenin signaling, and lipid metabolism in skeletal muscle, kidney, and heart [16, 42–45]. TGF-β plays an important role in fibrosis and is maintained in the extracellular matrix (ECM) in a latent state and must be activated before binding to its receptors [46–48]. In recent years, more attention has been paid to the TGF-β/Smad signaling pathway as an effective target for antifibrotic therapy [47]. p-Smad2/3 is the main downstream regulator that promotes TGF-β1-mediated tissue fibrosis, while Smad7 serves as a negative feedback regulator, thus protecting against TGF-β1-mediated fibrosis [48]. Smad7 is an intracellular antagonist of TGF-β signaling pathways and modulates muscle growth in vivo. Lack of Smad7 results in decreased muscle function and delayed post-injury tissue recovery/regeneration. Conversely, Smad2/3 signaling in the absence of muscle Smad7 results in reduced proliferation and differentiation of myoblasts [49]. Overexpression of Smad7 in vitro accelerates myoblast differentiation, whereas inhibition of Smad7 expression by small interfering RNA results in impaired differentiation [50]. Thus, the increase in Smad7 and decrease in Smad2 within days of injury in the B1KO group suggest that the reduced collagen deposition in areas of injury, and hence muscle fibrosis, is associated with inhibition of this pathway in injured tissue in the absence of B1R.

Wound contraction and tissue remodeling starts in the second week post-injury and represent the final stages of the healing process [51]. During this period, activation of the α-SMA gene transforms fibroblast into a highly contractile myofibroblast [52]. These myofibroblasts (MFs) exhibit, in addition to high contractile strength, a non-migratory characteristic and inhibit ECM production [53]. TGF-β1 is the main factor inducing the expression of α-SMA in MFs [52, 54], however in keeping with previous studies [55–57], the differentiation and regeneration of myofibers in skeletal muscle expresses α-SMA, and therefore this marker can be used in the quantification of myoblasts in in vivo sections. In our study, B2KO and B1B2KO animals showed exacerbated *Tgf-β1* expression but no increase in the number of α-SMA + cells. B1KO, in addition to expressing about twofold lower *Tgf-β1* compared to B2KO and B1B2KO animals, presented higher numbers of α-SMA + cells from the second week post-injury. Collectively these data suggest that the absence of B2R alone as also absence of B1R/B2R combined can disrupt the regulation of myoblast/myofibroblasts by *Tgf-β1* expression, but not prevent excessive fibrosis. Also, B1R can be targeted to serve both as a regulator of *Tgf-β1* expression and modulate the activation of these cells toward an improved fibrotic phenotype during musculoskeletal healing.

Regulatory T (Treg) cells expressing CD69 in injured skeletal muscle regulate the expansion of satellite cells (SCs), influencing the outcome of the regenerative process [33]. T cells have also been identified in dystrophic and injured skeletal muscle. However, their function was not established until recent studies focused on a specific population of Treg cells [58]. Activated T helper cells are able to infiltrate post-injury muscle tissue and exhibit characteristics of regulatory T cells, and alter TGF-β expression [59]. Tregs cells can be found in skeletal muscle and can promote tissue repair by acting directly on parenchymal cells [60], as well as promoting the transition from M1 to M2 in macrophages, but also by acting directly on muscle SCs [61]. Castiglioni et al. demonstrated that in the absence of T cells, muscle regeneration is compromised [59].

Studies have analyzed the ability of T cells to be activated and migrate after B1R blockade under different in vitro conditions in a neuroinflammation model. Several activation markers, including CD69, were not altered in CD4 T cells isolated from B1KO animals compared to WT controls. Consistent with these findings, no difference in the expression of adhesion molecules CD11a and CD49d became apparent in B1KO T cells and controls under inflammatory or in vitro conditions [62]. Other models such as experimental autoimmune encephalomyelitis (EAE) in mice have shown that in B1KO and B2KO animals or mice pretreated with B1R blocker (DALBK) and B2R antagonist (HOE-140), a decrease in the CD69 + T cell population occurs [63]. Our results suggest that, at least for injured skeletal muscle, the absence of the B2R may promote the excessive increase in the CD69 + cell population in the first day post-injury, with a subsequent decrease by week 2, while the absence of the B1R maintains this population at levels normally found in the pathophysiology of a normal animal for the first few days, followed by a major decrease relative to this group by week 2 post-injury.

Several studies have shown that macrophages are essential for skeletal muscle regeneration [28, 64, 65]; however, these cells are also implicated in the fibrosis of numerous tissues [66, 67]. The remarkable plasticity of this cell type explains, in part, their diverse functions in tissue repair [68], exhibiting diverse and often opposing phenotypes when exposed to different environmental stimuli. For example, although T cells can tolerate low oxygen levels in the early stages of inflammation, macrophages adapt quickly to these conditions and change their metabolism to anaerobic glycolysis [69]. The first macrophages recruited to areas of injury are so-called M1-type (pro-inflammatory) macrophages which, together with neutrophilic granulocytes, are responsible for phagocytosis of cellular debris. They secrete various cytokines, growth factors, and pro-inflammatory mediators (e.g., tumor necrosis factor-α (TNF-α), nitric oxide (NO), and interleukins, among them interleukin-6 (IL-6)) [69]. This macrophage subtype strongly impacts the microenvironment of the lesion, affecting the behavior of adjacent cells and stem cells in regeneration [66, 70]. Furthermore, it has been described that these cells stimulate IL-6-dependent myoblast proliferation [71]. Local production of IL-6 by skeletal muscle cells and stromal cells promotes activation of SCs, thus enhancing myotubule regeneration in the face of injury. Our data showed an excessive increase in CD69 + cells and IL-6 expression in the B2KO group on day 4 post-injury. However, the potentially beneficial effects of these changes did not improve the regenerative phenotype of these animals, showing that such effects are associated with B2R expression at least at basal levels. Soon after muscle injury, the number of neutrophilic granulocytes increased rapidly in the B2KO group, which may have contributed to the increased IL-6 expression. Although many studies need to be done in order to clarify the role of IL-6 in skeletal muscle, this result suggests that a high expression of this myokine may be anticipated in injured skeletal muscle in the absence of B2R, thus contributing to the exacerbated inflammatory response in the face of injury and hindering post-injury tissue resolution.

The angiogenic process and thus revascularization is crucial for the initiation of the cascade of regenerative events [72]. It is a sequence of complex physiological processes of vasodilation, basal membrane degradation, endothelial cell migration, chemotaxis, increased vascular permeability, and eventually endothelial cell proliferation and vessel formation [73]. The capillary system in skeletal muscle has the ability to adapt in a short period of time in structure and function to different environmental changes and therefore can be considered plastic [74]. Angiogenesis is an example of microcirculation plasticity, which refers to the amplification of the capillary system in response to an external stimulus, for example in muscles due to contraction and/or increased blood flow [75–77] or division, although the latter process is not yet fully elucidated. In general, VEGF is an angiogenic factor that is essential for skeletal muscle development [78] and also plays a role in the maintenance of capillaries in adult skeletal muscle [79]. We now know that for physiological angiogenesis to be successfully regulated, VEGF must work in concert with other pro-angiogenic and antiangiogenic factors [80]. We currently know that bradykinin promotes angiogenesis by positively regulating bFGF via the B1R or by stimulating VEGF formation via the B2R [81]. In our model, the absence of B1R, combined with the presence of B2R provides a pro-angiogenic niche for post-injury tissue resolution superior to WT animals, as shown in particular in the increased vascular density and expression of *Vegfa* and *Ncl*.

All SCs express the nodal transcription factor Pax7, which is fundamental for satellite cell maintenance and regenerative capacity in skeletal muscle [82–84]. Pax7 persists in newly activated proliferating SCs and is rapidly suppressed in cells that engage in terminal differentiation [85]. On the other hand, MyoD, along with other factors such as Myf5, are key regulators of skeletal muscle lineage determination in the embryo, and its expression is induced in SCs after muscle injury [86, 87]. MyoD is also expressed by SCs precursors in developing skeletal muscle, although its most effective contribution is found in injury-induced SCs [88–90]. The concomitant significant increase in Pax7 and MyoD expression on day 4 post-injury indicates that, at least in the B1KO group, SCs activity and myoblast differentiation were enhanced and accelerated. Our data also demonstrated the recovery ability of the B1KO group in strength endurance tests in resuming their post-injury performance, doing so in a shorter time when compared to the other experimental groups, which is directly associated with more efficient tissue repair.

As presented in the histomorphological section, the B2KO group showed an extensive area of injury with excessive inflammation. It is now well established that inflammation is directly linked to the loss of muscle contractile function due to increased levels of pro-inflammatory cytokines and chemokines, contributing to the muscle weakness seen after acute muscle injury [91, 92].

**Fig. 9:**
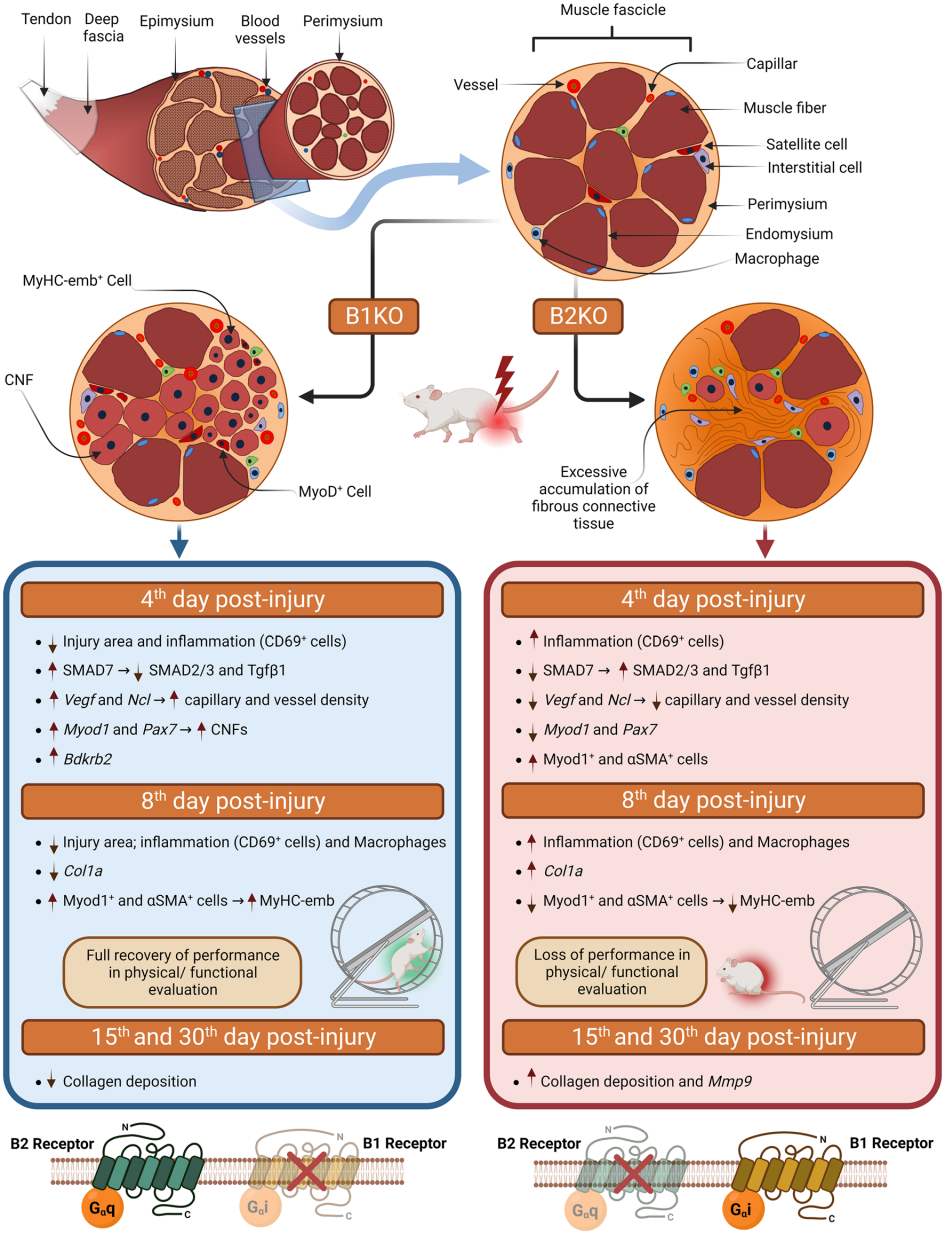
Scheme of the main differential effects observed in knockout animals for kinin Bdkrb1 and Bdkrb2 receptors. CNF, centronucleated fibers

The B1 and B2 kinin receptors have already been described in skeletal muscle [15, 93]. Although their specific labeling in tissue is still a challenge throughout the kinin study community, their gene expression has been explored in some studies that directly or indirectly involve skeletal muscle tissue [16, 94, 95]. Here we present for the first time the variation in kinin receptor expression in the days following acute skeletal muscle tissue injury. Figure 9 summarizes the main changes found in injured skeletal muscles when B1R or B2R are lacking. Although B1KO animals showed remarkable differences in the tissue repair process on almost every day after injury compared to B2KO and B1B2KO, the 4th day post-injury seems to have been a decisive moment for these differences. We highlight that the B2KO and B1B2KO groups showed similarity in almost all aspects tested, evidencing that the lack of B2R is a determining factor in the repair processes of this kind of injury.

Interestingly, B1KO animals also showed increased expression of *Bdkrb2* on the 4th day post-injury. This result may indicate that some of the reparative effects found in the B1KO animals may be related, not only to the absence of B1R, but rather, to the overexpression of B2R.

## Conclusion

This study demonstrates the capability of post-injury skeletal muscle tissue repair when kinin receptors are lacking. In addition, it was possible to show the differential effect for each of the receptors on the major steps of post-injury tissue repair. B1 knockout animals showed a remarkable tissue repair capacity with less fibrosis, including better performances in functional tests when compared to the other groups. On the other hand, the absence of B2 receptor alone or combined with the absence of B1 receptor led to intense tissue destruction and more fibrosis. To our knowledge, this is the first study to characterize post-injury musculoskeletal tissue events in knockout animals for kinin receptors and suggest that such receptors may represent novel therapeutic targets for the development of new therapies for musculoskeletal injuries and even myopathies in regenerative medicine and rehabilitation.

## Materials and methods

### Animal experimentation

For the experiments 12-week-old male kinin receptor knockout mice and WT were used on the C57Bl/6 background bred at the Department of Biophysics, Universidade Federal de São Paulo (UNIFESP). The genetic alteration in the mice lacking B1 receptor (*Bdkrb1* -/- or B1KO), B2 receptor (*Bdkrb2* -/- or B2KO) and both receptors (*Bdkrb1* -/- *Bdkrb2* -/- or B1B2KO) were previously described [96–98]. Details of the genotyping of kinin B1 and B2 knockout mice and muscle contusion model are provided in SI Experimental Procedures (Fig. S1).

### Skeletal muscle histomorphology and immunofluorescence microscopy

Mice were euthanized by cervical dislocation to collect TA muscles. Muscles were quickly dissected for cryosectioning, frozen in isopentane cooled in liquid nitrogen and stored at − 80 °C until processing. For the analyzes in paraffined sections, we follow the protocols described previously [99]. Antibodies are listed in Table S2. Details are provided in SI Experimental Procedures.

### RNA Preparation and RT-PCR

Mice were euthanized by cervical dislocation to collect TA muscles. Total RNA from TA muscles was extracted using RNeasy^®^ Fibrous Tissue Mini Kit (Qiagen, Cat# 74704), and the cDNA was synthesized using RT2 First Strand Kit (Qiagen). In order to amplify the gene transcripts, the primers listed in Table S3 were used in the SYBR Green or TaqMan system on the device of The Applied Biosystems^®^ QuantStudio^®^ 5 Real-Time PCR System (Thermo Fisher, USA). *Gapdh* and *Actb* gene were used as internal control. Each biological sample had a single replicate reaction, and two biological samples were used for all of them. Relative gene expression was calculated by 2-ΔΔCT. The changes in mRNA expression were expressed as fold changes relative to control. Details are provided in SI Experimental Procedures.

### Functional tests assessment

Treadmill running ability and climbing strength/ endurance test combined with incremental mass series were tested in all groups in the functional analyzes. Details are provided in SI Experimental Procedures.

### Statistical analysis

All statistical analysis was performed in GraphPad Prism (GraphPad Software). Data analysis was performed using one-way ANOVA and post hoc Tukey correction or Student’s *t* test assuming two-tailed distribution and unequal variances. The use of an asterisk or octothorpe denotes statistical significance of *P* < 0.05 between the sample groups described. In graphs with more than two groups, statistical analysis describes the two groups connected by a bar line. All results are shown as mean ± SD unless stated otherwise. All samples represent at least *n* = 5.

## Supplementary Information

Below is the link to the electronic supplementary material.

Supplementary file1 (DOCX 9985 KB)

## Data Availability

All data generated during and/or analyzed during this study are included in this published article and its supplementary information. Any detailed data supporting the findings of this study are available from the corresponding authors upon reasonable request.

## Abbreviations

ACE: Angiotensin-converting enzyme
B1B2KO: B1 and B2 receptor double knockout mice
B1KO: B1 receptor knockout mice
B2KO: B2 receptor knockout mice
BDKRB1 or B1R: B1 bradykinin receptor
BDKRB2 or B2R: B2 bradykinin receptor
BK: Bradykinin
CNFs: Center nucleated fibers
COL1A: Collagen Type I Alpha 1 Chain
DAPI: 4’,6-Diamidino-2-phenylindole
ECM: Extracellular matrix
HE: Hematoxylin–eosin staining
HPF: High-power fields
IL-6: Interleukin-6
KKS: Kallikrein-kinin system
MFs: Myofibroblasts
Myf5: Myogenic Factor 5
MyHC-emb: Embryonic/developmental myosin heavy chain
MyoD: Myogenic differentiation 1
NO: Nitric oxide
Pax7: Nodal transcription factor Pax7
p-SMAD2/3: Phosphorylated Smad2/3
RAS: Renin-angiotensin system
SCs: Satellite cells
SMAD2: SMAD Family Member 2
SMAD7: SMAD Family Member 7
TA: Tibialis anterior
Tgfβ1: Transforming growth factor beta 1
TNF-α: Tumor necrosis factor-α
Treg: Regulatory T cells
VEGF: Vascular endothelial growth factor
WT: Wild type mice
αSMA: Alpha-smooth muscle actin

## References

[1] Yelin E, Weinstein S, King T (2019). An update on the burden of musculoskeletal diseases in the U.S. Semin Arthritis Rheum.

[2] Järvinen TAH, Järvinen TLN, Kääriäinen M, Kalimo H, Järvinen M (2005). Muscle injuries: biology and treatment. Am J Sport Med.

[3] Ekstrand J, Hägglund M, Waldén M (2011). Injury incidence and injury patterns in professional football: the UEFA injury study. Br J Sports Med.

[4] Hägglund M, Waldén M, Ekstrand J (2013). Risk factors for lower extremity muscle injury in professional soccer: the UEFA injury study. Am J Sports Med.

[5] Orchard J, Best TM (2002). The management of muscle strain injuries: an early return versus the risk of recurrence. Clin J Sport Med.

[6] Järvinen TAH, Järvinen M, Kalimo H (2013). Regeneration of injured skeletal muscle after the injury. Muscles Ligaments Tendons J.

[7] Tidball JG (2005). Inflammatory processes in muscle injury and repair. Am J Physiol Regul Integr Comp Physiol.

[8] Tsuji S, Taniuchi S, Hasui M, Yamamoto A, Kobayashi Y (2002). Increased nitric oxide production by neutrophils from patients with chronic granulomatous disease on trimethoprim-sulfamethoxazole. Nitric Oxide Biol Chem.

[9] Webb NJA, Myers CR, Watson CJ, Bottomley MJ, Brenchley PEC (1998). Activated human neutrophils express vascular endothelial growth factor (vegf). Cytokine.

[10] Kharraz Y, Guerra J, Pessina P, Serrano AL, Muñoz-Cánoves P (2014). Understanding the process of fibrosis in duchenne muscular dystrophy. Biomed Res Int.

[11] Stilhano RS (2017). Reduction in skeletal muscle fibrosis of spontaneously hypertensive rats after laceration by microRNA targeting angiotensin II receptor. PLoS ONE.

[12] Murphy AM, Wong AL, Bezuhly M (2015). Modulation of angiotensin II signaling in the prevention of fibrosis. Fibrogenes Tissue Repair.

[13] Bhoola KD, Figueroa CD, Worthy K (1992). Bioregulation of kinins: Kallikreins, kininogens, and kininases. Pharmacol Rev.

[14] Madeddu P, Emanueli C, El-Dahr S (2007). Mechanisms of disease: the tissue kallikrein-kinin system in hypertension and vascular remodeling. Nat Clin Pract Nephrol.

[15] Figueroa CD, Dietze G, Muller-Esterl W (1996). Immunolocalization of bradykinin B2 receptors on skeletal muscle cells. Diabetes.

[16] Acuña MJ (2018). Blockade of Bradykinin receptors worsens the dystrophic phenotype of mdx mice: differential effects for B1 and B2 receptors. J Cell Commun Signal.

[17] Leeb-Lundberg LMF, Marceau F, Müller-Esterl W, Pettibone DJ, Zuraw BL (2005). International union of pharmacology. XLV. Classification of the kinin receptor family: from molecular mechanisms to pathophysiological consequences. Pharmacol Rev.

[18] Qadri F, Bader M (2018). Kinin B1 receptors as a therapeutic target for inflammation. Expert Opin Ther Targets.

[19] Yao YY, Yin H, Shen B, Chao L, Chao J (2007). Tissue Kallikrein and Kinin infusion rescues failing myocardium after myocardial infarction. J Card Fail.

[20] Sancho-Bru P (2007). Bradykinin attenuates hepatocellular damage and fibrosis in rats with chronic liver injury. Gastroenterology.

[21] Kakoki M (2010). Lack of both bradykinin B1 and B2 receptors enhances nephropathy, neuropathy, and bone mineral loss in Akita diabetic mice. Proc Natl Acad Sci USA.

[22] Liu Y (2010). Blockade of endogenous tissue kallikrein aggravates renal injury by enhancing oxidative stress and inhibiting matrix degradation. Am J Physiol Ren Physiol.

[23] Emanueli C, et al*.* Receptor knockout mice. 1999;2359–2366.

[24] Schanstra JP (2002). In vivo bradykinin B2 receptor activation reduces renal fibrosis. J Clin Invest.

[25] Chao J, Shen B, Gao L, Xia CF, Bledsoe G, Chao L (2010). Tissue kallikrein in cardiovascular, cerebrovascular and renal diseases and skin wound healing. Biol Chem.

[26] Tu L (2008). Delivery of recombinant adeno-associated virus-mediated human tissue kallikrein for therapy of chronic renal failure in rats. Hum Gene Ther.

[27] Zhu D, Zhang L, Cheng L, Ren L, Tang J, Sun D (2016). Pancreatic kininogenase ameliorates renal fibrosis in streptozotocin induced-diabetic nephropathy rat. Kidney Blood Press Res.

[28] Novak EW-HTKML (2014). Macrophage activation and skeletal muscle healing following traumatic injury. J Pathol.

[29] Berton G, Gordon S (1983). Modulation of macrophage mannosyl-specific receptors by cultivation on immobilized zymosan. Effects on superoxide-anion release and phagocytosis. Immunology.

[30] Lee F (1985). Isolation of cDNA for a human granulocyte-macrophage colony-stimulating factor by functional expression in mammalian cells. Proc Natl Acad Sci USA.

[31] Springer ML, Ozawa CR, Blau HM (2002). Transient production of α-smooth muscle actin by skeletal myoblasts during differentiation in culture and following intramuscular implantation. Cell Motil Cytoskeleton.

[32] Mondrinos MJ (2021). Surface-directed engineering of tissue anisotropy in microphysiological models of musculoskeletal tissue. Sci Adv.

[33] Schiaffino S, Pereira MG, Ciciliot S, Rovere-Querini P (2017). Regulatory T cells and skeletal muscle regeneration. FEBS J.

[34] Chen X (2005). Dedifferentiation of adult human myoblasts induced by ciliary neurotrophic factor in vitro. Mol Biol Cell.

[35] Kuang S, Kuroda K, Le Grand F, Rudnicki MA (2007). Asymmetric self-renewal and commitment of satellite stem cells in muscle. Cell.

[36] Dalbis A, Couteaux R, Janmot C, Roulet A, Mira J-C (1988). Regeneration after cardiotoxin injury of innervated and denervated slow and fast muscles of mammals. Myosin isoform analysis. Eur J Biochem.

[37] Cervelli M, Leonetti A, Duranti G, Sabatini S, Ceci R, Mariottini P (2018). Skeletal muscle pathophysiology: the emerging role of spermine oxidase and spermidine. Med Sci.

[38] Schiaffino S, Rossi AC, Smerdu V, Leinwand LA, Reggiani C (2015). Developmental myosins: expression patterns and functional significance. Skeletal Muscle.

[39] Chao J (2007). Kinin infusion prevents renal inflammation, apoptosis, and fibrosis via inhibition of oxidative stress and mitogen-activated protein kinase activity. Hypertension.

[40] Guevara-Lora I (2012). Kinin-mediated inflammation in neurodegenerative disorders. Neurochem Int.

[41] Hachana S, Bhat M, Sénécal J, Huppé-Gourgues F, Couture R, Vaucher E (2018). Expression, distribution and function of kinin B1 receptor in the rat diabetic retina. Br J Pharmacol.

[42] Delaney K, Kasprzycka P, Ciemerych MA, Zimowska M (2017). The role of TGF-b1 during skeletal muscle regeneration. Cell Biol Int.

[43] Ábrigo J (2018). TGF-β requires the activation of canonical and non-canonical signalling pathways to induce skeletal muscle atrophy. Biol Chem.

[44] Cabello-Verrugio C, Rivera JC, Garcia D (2017). Skeletal muscle wasting: new role of nonclassical renin-angiotensin system. Curr Opin Clin Nutr Metab Care.

[45] Chen JL, Colgan TD, Walton KL, Gregorevic P, Harrison CA. The TGF-β signalling network in muscle development, adaptation and disease. In: Advances in experimental medicine and biology, vol. 900. Springer New York LLC; 2016. p. 97–131. 10.1007/978-3-319-27511-6_5 27003398

[46] Biernacka A, Dobaczewski M, Frangogiannis NG (2011). TGF-β signaling in fibrosis. Growth Factors.

[47] Walton KL, Johnson KE, Harrison CA (2017). Targeting TGF-β mediated SMAD signaling for the prevention of fibrosis. Front Pharmacol.

[48] Hu HH (2018). New insights into TGF-β/Smad signaling in tissue fibrosis. Chem Biol Interact.

[49] Cohen TV, Kollias HD, Liu N, Ward CW, Wagner KR (2015). Genetic disruption of Smad7 impairs skeletal muscle growth and regeneration. J Physiol.

[50] Kollias HD, Perry RLS, Miyake T, Aziz A, McDermott JC (2006). Smad7 promotes and enhances skeletal muscle differentiation. Mol Cell Biol.

[51] Järvinen TAH, Järvinen TLN, Kääriäinen M, Kalimo H, Järvinen M (2005). Muscle injuries: biology and treatment. Am J Sports Med.

[52] Desmouliere A, Geinoz A, Gabbiani F, Gabbiani G (1993). Transforming growth factor-β1 induces α-smooth muscle actin expression in granulation tissue myofibroblasts and in quiescent and growing cultured fibroblasts. J Cell Biol.

[53] Gabbiani G (2021). 50 years of myofibroblasts: how the myofibroblast concept evolved. Methods Mol Biol.

[54] Vallée A, Lecarpentier Y. TGF-β in fibrosis by acting as a conductor for contractile properties of myofibroblasts. 10.1186/s13578-019-0362-3 PMC6902440 31827764

[55] Babai F, Musevi-Aghdam J, Schurch W, Royal A, Gabbiani G (1990). Coexpression of alpha-sarcomeric actin, alpha-smooth muscle actin and desmin during myogenesis in rat and mouse embryos I. Skeletal muscle. Differentiation.

[56] Springer ML, Ozawa CR, Blau HM (2002). Transient production of alpha-smooth muscle actin by skeletal myoblasts during differentiation in culture and following intramuscular implantation. Cell Motil Cytoskeleton.

[57] Murray IR (2017). αv integrins on mesenchymal cells regulate skeletal and cardiac muscle fibrosis. Nat Commun.

[58] Wu J, Ren B, Wang D, Lin H (2022). Regulatory T cells in skeletal muscle repair and regeneration: recent insights. Cell Death Dis.

[59] Castiglioni A (2015). FOXP3+ T cells recruited to sites of sterile skeletal muscle injury regulate the fate of satellite cells and guide effective tissue regeneration. PLoS ONE.

[60] Panduro M, Benoist C, Mathis D (2016). Tissue tregs. Annu Rev Immunol.

[61] Burzyn D (2013). A special population of regulatory T cells potentiates muscle repair. Cell.

[62] Göbel K (2011). Blockade of the kinin receptor B1 protects from autoimmune CNS disease by reducing leukocyte trafficking. J Autoimmun.

[63] Dutra RC (2011). The role of kinin receptors in preventing neuroinflammation and its clinical severity during experimental autoimmune encephalomyelitis in mice. PLoS ONE.

[64] Arnold L (2007). Inflammatory monocytes recruited after skeletal muscle injury switch into antiinflammatory macrophages to support myogenesis. J Exp Med.

[65] Martins L (2020). Skeletal muscle healing by M1-like macrophages produced by transient expression of exogenous GM-CSF. Stem Cell Res Ther.

[66] Villalta SA, Rinaldi C, Deng B, Liu G, Fedor B, Tidball JG (2011). Interleukin-10 reduces the pathology of mdx muscular dystrophy by deactivating M1 macrophages and modulating macrophage phenotype. Hum Mol Genet.

[67] Wynn TA, Vannella KM (2016). Macrophages in tissue repair, regeneration, and fibrosis. Immunity.

[68] Sass FA (2018). Immunology guides skeletal muscle regeneration. Int J Mol Sci.

[69] Zhu L, Zhao Q, Yang T, Ding W, Zhao Y (2015). Cellular metabolism and macrophage functional polarization. Int Rev Immunol.

[70] Tidball JG, Villalta SA (2010). Regulatory interactions between muscle and the immune system during muscle regeneration. Am J Physiol Regul Integr Compar Physiol.

[71] Zhang C, Li Y, Wu Y, Wang L, Wang X, Du J (2013). Interleukin-6/signal transducer and activator of transcription 3 (STAT3) pathway is essential for macrophage infiltration and myoblast proliferation during muscle regeneration. J Biol Chem.

[72] Borselli C (2010). Functional muscle regeneration with combined delivery of angiogenesis and myogenesis factors. Proc Natl Acad Sci USA.

[73] Pandya NM, Dhalla NS, Santani DD (2006). Angiogenesis—a new target for future therapy. Vascul Pharmacol.

[74] Hudlicka O (2011). Microcirculation in skeletal muscle. Muscles Ligaments Tendons J.

[75] Olfert IM, Baum O, Hellsten Y, Egginton S (2016). Advances and challenges in skeletal muscle angiogenesis. Am J Physiol Circ Physiol.

[76] Zhou AL, Egginton S, Brown MD, Hudlická O (1998). Capillary growth in overloaded, hypertrophic adult rat skeletal muscle: an ultrastructural study. Anat Rec.

[77] Ebina T (2002). Physiological angiogenesis in electrically stimulated skeletal muscle in rabbits: characterization of capillary sprouting by ultrastructural 3-D reconstruction study. Pathol Int.

[78] Gerber HP, Vu TH, Ryan AM, Kowalski J, Werb Z, Ferrara N (1999). VEGF couples hypertrophic cartilage remodeling, ossification and angiogenesis during endochondral bone formation. Nat Med.

[79] Tang K, Breen EC, Gerber HP, Ferrara NMA, Wagner PD (2004). Capillary regression in vascular endothelial growth factor-deficient skeletal muscle. Physiol Genomics.

[80] Olfert IM, Birot O (2011). Importance of anti-angiogenic factors in the regulation of skeletal muscle angiogenesis. Microcirculation.

[81] Colman R (2006). Regulation of angiogenesis by the Kallikrein-Kinin system. Curr Pharm Des.

[82] Von Maltzahn J, Jones AE, Parks RJ, Rudnicki MA (2013). Pax7 is critical for the normal function of satellite cells in adult skeletal muscle. Proc Natl Acad Sci USA.

[83] Sambasivan R (2011). Pax7-expressing satellite cells are indispensable for adult skeletal muscle regeneration. Development.

[84] Lepper C, Partridge TA, Fan CM (2011). An absolute requirement for Pax7-positive satellite cells in acute injury-induced skeletal muscle regeneration. Development.

[85] Zammit PS, Golding JP, Nagata Y, Hudon V, Partridge TA, Beauchamp JR (2004). Muscle satellite cells adopt divergent fates: a mechanism for self-renewal?. J Cell Biol.

[86] Rudnicki MA, Schnegelsberg PNJ, Stead RH, Braun T, Arnold HH, Jaenisch R (1993). MyoD or Myf-5 is required for the formation of skeletal muscle. Cell.

[87] Yin H, Price F, Rudnicki MA (2013). Satellite cells and the muscle stem cell niche. Physiol Rev.

[88] Kuang S, Rudnicki MA (2008). The emerging biology of satellite cells and their therapeutic potential. Trends Mol Med.

[89] Bentzinger CF, Von Maltzahn J, Rudnicki MA (2010). Extrinsic regulation of satellite cell specification. Stem Cell Res Ther.

[90] Yamamoto M (2018). Loss of MyoD and Myf5 in skeletal muscle stem cells results in altered myogenic programming and failed regeneration. Stem Cell Reports.

[91] Pinniger GJ, Lavin T, Bakker AJ (2012). Skeletal muscle weakness caused by carrageenan-induced inflammation. Muscle Nerve.

[92] Mori T (2018). Post-injury stretch promotes recovery in a rat model of muscle damage induced by lengthening contractions. J Physiol Sci.

[93] Emanueli C (2002). Prevention of diabetes-induced microangiopathy by human tissue kallikrein gene transfer. Circulation.

[94] Reis FCG (2015). Deletion of Kinin B2 receptor alters muscle metabolism and exercise performance. PLoS ONE.

[95] Parreiras-e-Silva LT (2014). The kinin B1 receptor regulates muscle-specific E3 ligases expression and is involved in skeletal muscle mass control. Clin Sci.

[96] Pesquero JB (2000). Hypoalgesia and altered inflammatory responses in mice lacking kinin B1 receptors. Proc Natl Acad Sci USA.

[97] Borkowski JA (1995). Targeted disruption of a B2 bradykinin receptor gene in mice eliminates bradykinin action in smooth muscle and neurons. J Biol Chem.

[98] Cayla C (2007). Mice deficient for both kinin receptors are normotensive and protected from endotoxin-induced hypotension. FASEB J.

[99] Martins L, Martin PKM, Han SW (2014). Angiogenic properties of mesenchymal stem cells in a mouse model of limb ischemia. Methods Mol Biol.

