# Supplementary material for: Kinin receptors regulate skeletal muscle regeneration: differential effects for B1 and B2 receptors
Source: Inflamm Res. 2023 Jul 18;72(8):1583–601. doi: 10.1007/s00011-023-01766-4 (PMC10499706; doi:10.1007/s00011-023-01766-4)
Supplement: Supplementary file 1 — Supplementary file1 (DOCX 9985 KB) [file 11_2023_1766_MOESM1_ESM.docx]

**Supporting Information for**

Kinin receptors regulate skeletal muscle regeneration: differential effects for B1 and B2 receptors.

Leonardo Martins^1,2,3*^; Weslley Wallace Amorim^2^; Marcos Fernandes Gregnani^4^; Ronaldo de Carvalho Araújo^4^; Fatimunnisa Qadri^5^; Michael Bader^5,6,7,8^; João Bosco Pesquero^2,9*^

^1^Division of Medical Sciences, Institute of Medical Biology of Polish Academy of Sciences (IMB-PAN), 3a Tylna St., 90-364, Łódź, Poland.
^2^Center for Research and Molecular Diagnosis of Genetic Diseases, Federal University of São Paulo, Rua Pedro de Toledo 669, 9th floor, 04039032, São Paulo, Brazil.

^3^Department of Biochemistry and Molecular Biology, Federal University of São Paulo, Rua Três de Maio 100, 4th floor, 04044-020, São Paulo, Brazil.
^4^Laboratory of Exercise Genetics and Metabolism, Federal University of São Paulo, Rua Pedro de Toledo 669, 9th floor, 04039032, São Paulo, Brazil.

^5^Max-Delbrück Center for Molecular Medicine (MDC), Robert-Rössle-Str. 10, 13125, Berlin, Germany.
^6^Institute for Biology, University of Lübeck, Ratzeburger Allee 160, 23562, Lübeck, Germany.
^7^Charité University Medicine Berlin, Charitéplatz 1, 10117, Berlin, Germany.
^8^German Center for Cardiovascular Research (DZHK), Potsdamer Str. 58, 10785, Berlin, Germany.
^9^Department of Biophysics, Federal University of São Paulo, Rua Botucatu 862, 6th floor, 04023-062, São Paulo, Brazil.

***Corresponding Authors:** Leonardo Martins and João Bosco Pesquero

**Emails:**  lmartin@cbm.pan.pl; [jbpesquero@unifesp.br](mailto:jbpesquero@unifesp.br)

**DOI:** 10.1007/s00011-023-01766-4

**Supporting Information Text**

**Genotyping of kinin B1 and B2 knockout mice and *in vivo* injury model**

Genomic DNA was isolated from a mouse tail fragment using an NaOH extraction protocol[1]. Kinin B1 and B2 receptor genes were amplified by polymerase chain reaction (PCR) using the following primers: B1 receptor (NM_007539.2) sense 5′-TGAAGCTGTGAGCTCTTTG-3′ and antisense 5′-GCTACCCAGATGAGCAGGCA-3′; B2 (NM_009747.2) receptor sense 5′-TGTCCTCAGCGTGTTCTTCC-3′ and antisense 5′-GGTCCTGAACACCAACATGG-3′ (**Figure** S1).

Animals were allowed to adapt to the laboratory for at least 1h before testing and were used only once. The experiments were conducted in accordance with the ethical principles established by the Brazilian College of Animal Experimentation (COBEA) and approved by the Ethics Committee for the use of animals (CEUA) of the Universidade Federal de São Paulo (Nº 6524250215). Food and water were supplied ad libitum and the animals were kept in a 12 h light/dark cycle and in a temperature-controlled room (22 ± 2° C).

Muscle contusion was performed based on the method previously described [2,3]. C57Bl/6 mice aging 10–12 weeks and weighting 25 g were anesthetized with an IP injection of ketamine (40 mg/kg of animal) and xylazine (10 mg/kg of animal). The mouse was positioned in ventral decubitus and the posterior limbs positioned extending the knee and plantar flexion. A stainless-steel ball weighting 16.2 g with 1.6 cm diameter was placed on the top of a PVC tube with 100 cm height to fall by gravity. At the bottom of the tube, a blunt-tipped nail (tip area is approximately equal to the TA muscle surface) was placed so that the impact was transferred to the entire TA muscle area (**Figure** S1). During first 4 days after contusion, fentanyl (0.05 mg/kg; IP) was administered to alleviate pain. Nonsteroidal anti-inflammatory drugs (NSAIDs) were not used here because these drugs affect muscle regeneration[4].

**Histological Evaluation and Analysis.** After the mice are euthanized 0, 4, 8, 15 or 30 days after injury, bilateral TA muscles were instantly frozen in isopentane (precooled in liquid nitrogen) and stored at -80°C until sectioned. Serial cryosections of 8-mm thickness were prepared (CryoStar™ NX70 Cryostat - Thermo Fisher Scientific) for immunostaining according to the protocol of **Table** S1. All images were captured inside injured areas and 10 randomly selected 200x or 400x high-powered fields (HPF) from each sample as previously described[3], [5]–[8]. The percentages of cells expressing each marker of the **Table** S2 was obtained after image acquisition with a fluorescence microscope (Keyence, Osaka, Japan; BZ-9000) and quantified using ImageJ (Rasband, W.S., ImageJ, U. S. National Institutes of Health, Bethesda, Maryland, USA) or ImagePro Plus (Media Cybernetics, Rockville, MD, USA) according to a previously described protocol[7], [9]–[12]. The results from the control group and each of the KO groups were compared.

**Sirius red staining combined with polarized light microscopy*.*** Collagen fibers are anisotropic and therefore birefringent. Picrosirius Red binds to collagen and enhances its natural birefringence. Placing the TA section stained with PicroSirius Red (Sirius red F3B (80115, Klinipath) between two polarization filters with 90 degrees of rotational difference between both filters allows clear identification of birefringent fibrils. Representative images of the injury areas stained with Sirius Red in all experimental groups are shown in **Figure** S3 along with Col1A labeling by immunofluorescence highlighting the borders of the injury zones.

**RNA Isolation and quantitative RT-PCR.** Injury or trauma alter the patterns of gene expression in tissues. The purpose of this procedure is to obtain total cellular RNA from mouse muscle for subsequent analysis of mRNA populations, either by the polymerase chain reaction (following reverse transcription). The protocol is based on a commercial preparation, RNeasy® Fibrous Tissue Mini Kit (Qiagen, Cat# 74704). The initial processing of the tissues requires a mechanical homogenization in the Polytron Tissue Homogenizer (Glen Mills, USA. A proteinase K digestion follows and nucleic acids are removed by the addition of ethanol, followed by application to a RNeasy column that retains the RNA and washes away the contaminants. RNA is then eluted with distilled water. In order to amplify the gene transcripts, the primers listed in **table** S3 were used in the SYBR Green or TaqMan system on the device of The Applied Biosystems® QuantStudio® 5 Real-Time PCR System (Thermo Fisher, USA).

**Endurance Stress Test.** The mice were subjected to five separate endurance stress tests over the course of the study to indirectly assess improvements in aerobic capacity with exercise (a test in the basal condition (day zero) and in the days following the injury). Animals from all groups were placed in individual lanes on the treadmill (Columbus Instruments, Columbus, OH, USA) and allowed to acclimatize for 30 min to eliminate any confounding effects due to stress or anxiety related to a new environment. The test began with a 5-min warm-up session at 5 m/min, followed by 1-m/min increases in speed every 1 min until the mouse reached exhaustion. A low-intensity electrically stimulus was provided to ensure compliance. Time to exhaustion (in min) was recorded when the mouse sat at the lower end of the treadmill, near a shock bar, for >10 s and was unresponsive to further stimulation to continue running.

**Ladder climbing test.** The climbing training protocol was adapted from Hornberger and Farrar[13]. For all the groups, ladder climbing test was conducted by using 1 m ladder with 1.0 cm grids. The ladder was set to attain 85-degree angle with the ground. One week adaptation was conducted by letting mice to climb up the ladder without any resistance. To reduce stress, outsource stimulus such as food reward and electrical stimulation were not given to the mice during the ladder climbing exercise. The mice were positioned at the very bottom of 1 meter ladder and motivated to climb up the ladder. When mice reached the very top of the ladder, 2-minute rest was given before the next trail of ladder climbing. After the one-week adaptation, resistance at 10 % of body weight was given to the mice by adding weight on the tail. The loads were increased gradually as the exercise sessions proceeded. To progressively increase exercise intensity, 3 grams of additional weights were applied after four successful trails.

**SI Figures**

**Figure S1.** Representative genotyping of kinin *Bdkrb1* and *Bdkrb2* receptor in all mouse groups (Top). The scheme of the *in vivo* injury model (Bottom). Apparatus height of 100 cm used for the transfer of the projectile weighing 16.2 g (A) to the tibialis anterior (TA) muscle of the animal placed in a supine position (B). Representative image of an injured leg after 24 hours (C). Division of animal groups: wild-type mice - C57BL/6 strains (WT), kinin B1 and B2 receptor knockout mice (B1KO, *Bdkrb1*-/- and B2KO, *Bdkrb2*-/-) and double knockout mice (B1B2KO, *Bdkrb1*-/- *Bdkrb2*-/-), indicating the time of study for groups (D). *N*=10/group.

**
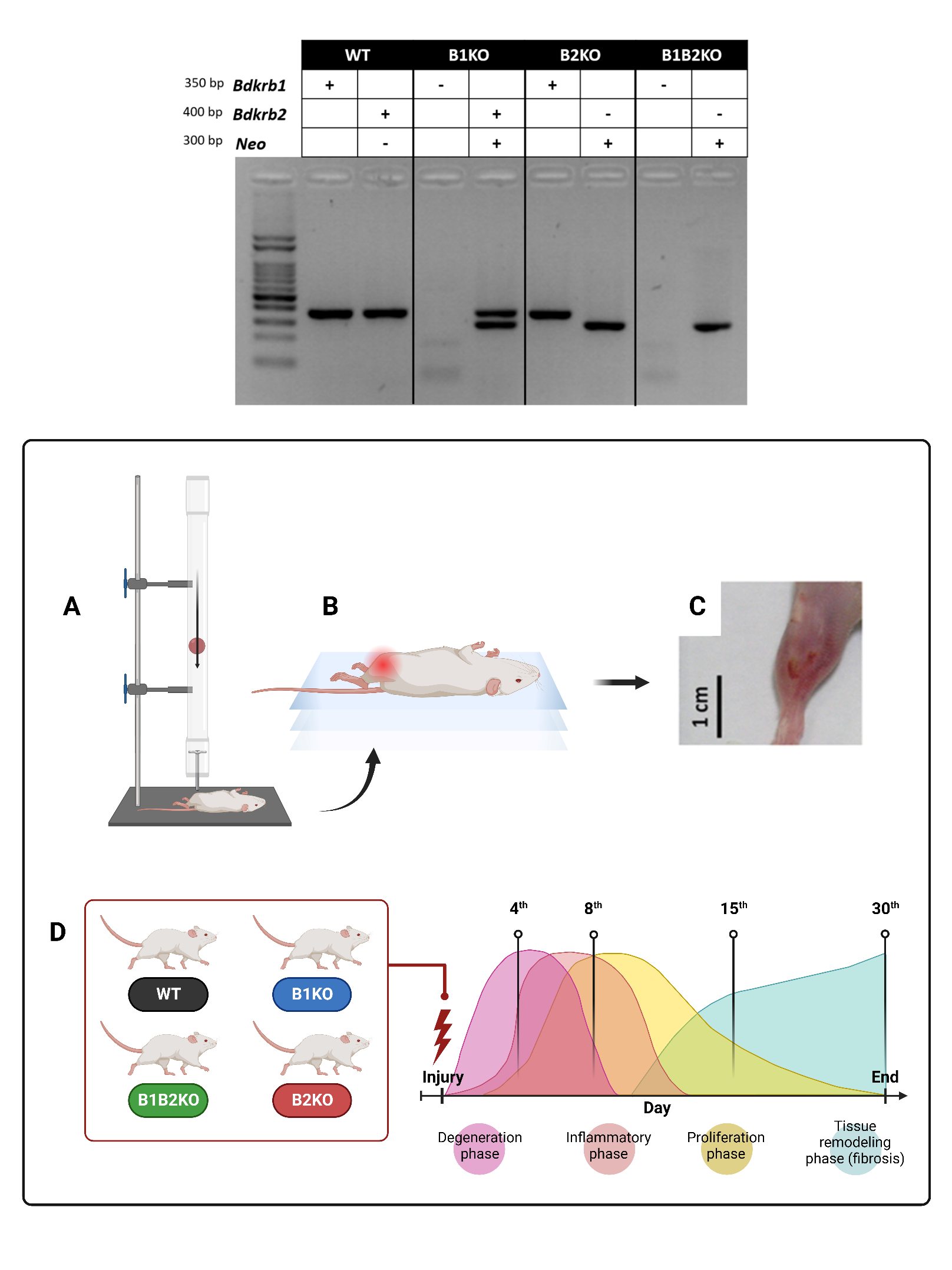
**

**Figure S2.** Differential macroscopic evaluation for B1 and B2KO mice. Macroscopic evaluation of representative injury in the skeletal muscle at 4, 8, and 15 days after injury and non-injured muscle (0 day) in each group. Animals from the B2KO group (C, G, L, and P) presented a worsening of the repair process and had a marked fibrotic area on the 8^th^ and 15^th^ (white arrow) post-injury days compared to the B1KO (B, F, J, and O) and WT animals (A, E, I, and N).


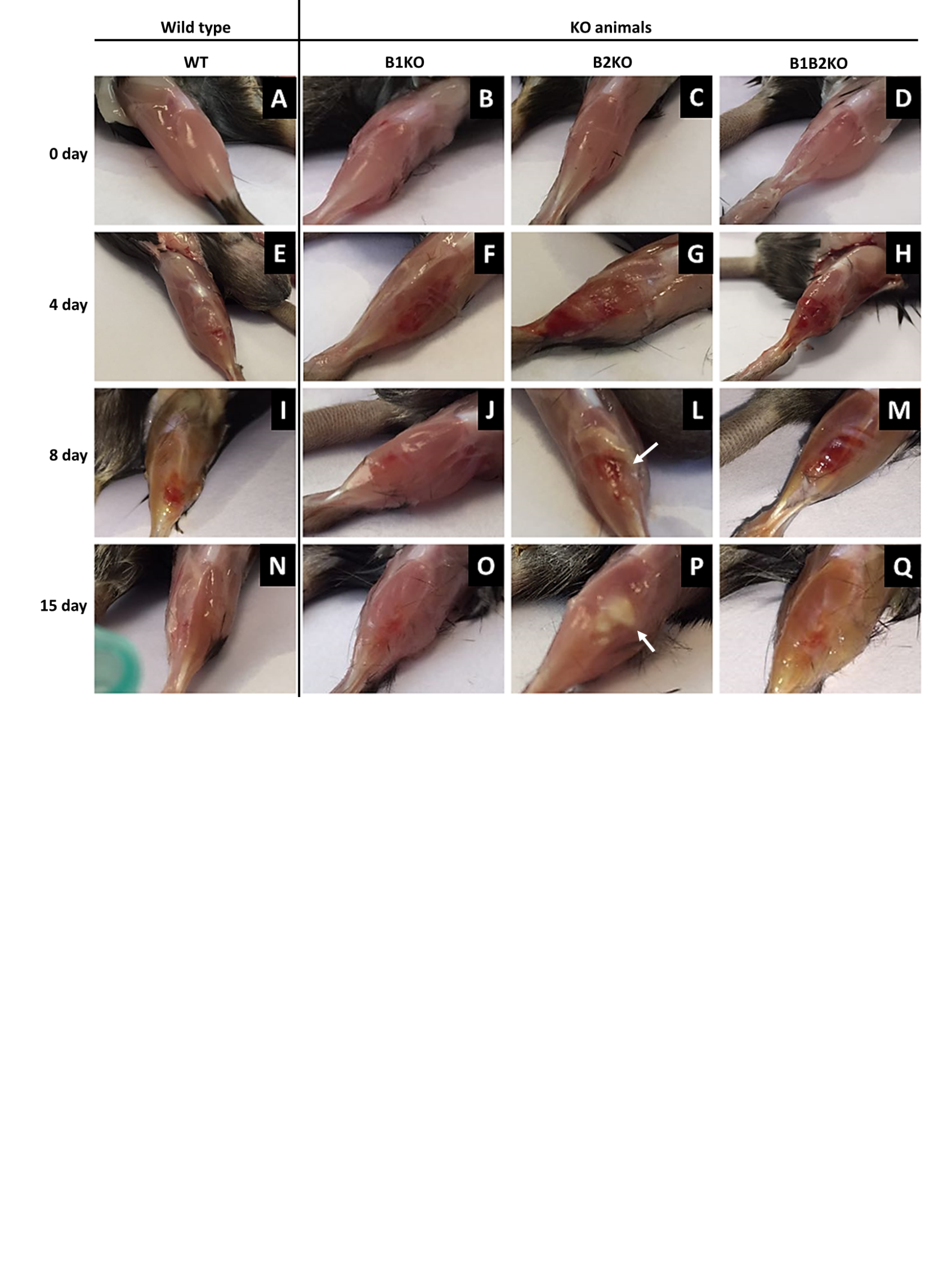


**Figure S3.** Representative images of the injury areas stained with Sirius Red**.** (A) Representative Sirius red staining’s at 4, 8, 15 and 30 days after injury in each group (Scale bars = 100 μm.) (B) Representative Sirius red staining combined with polarized light microscopy for collagen fiber structure. Yellow-red strong birefringence (type I collagen) and greenish color with thinner fibers (type III collagen) (white arrow) at 4, 8, 15 and 30 days after injury in each group (Scale bars = 100 μm.) (C) Representative immunofluorescence analyses of Col1A in boundary areas found in injury zones of the TA skeletal muscle. Col1A (red) DAPI, 4’,6-diamidino-2-phenylindole stain (blue). (Scale bars = 100 μm.) Perimysium (arrow); injury area (asterisk); border (line).

**
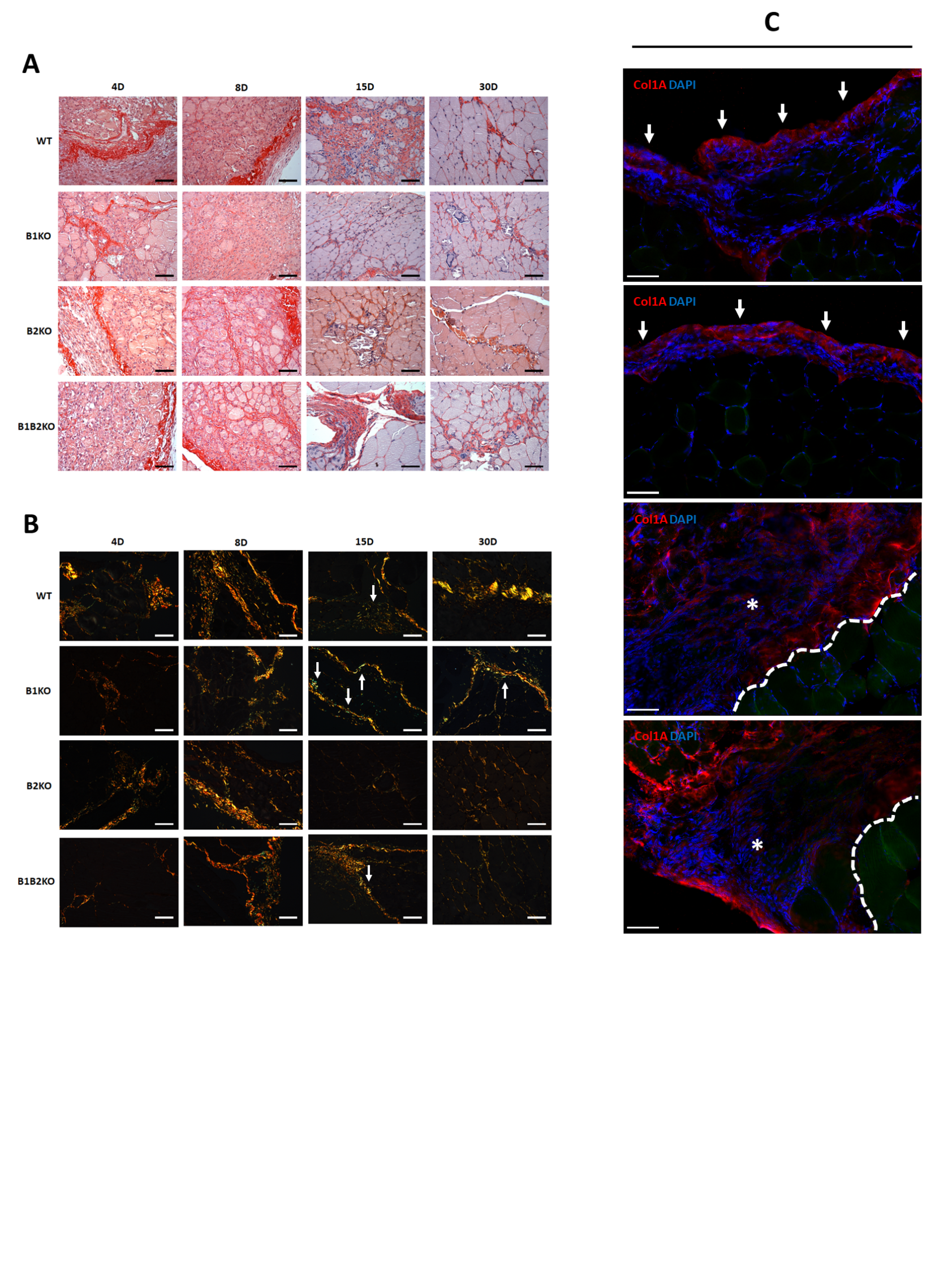
**

**Figure S4.** αSMA-expressing myoblast/ muscle precursor cells in injured area. Representative immunofluorescence analyses alpha-smooth muscle actin (αSMA) in the injured skeletal muscle in each group (A) and graphical comparison of the number of αSMA-expressing myofibroblasts in each group (B). Area of injury trapping blood vessels (arrowhead) and myoblasts (arrow). (*,0.05). DAPI, 4’,6-diamidino-2-phenylindole stain (blue).


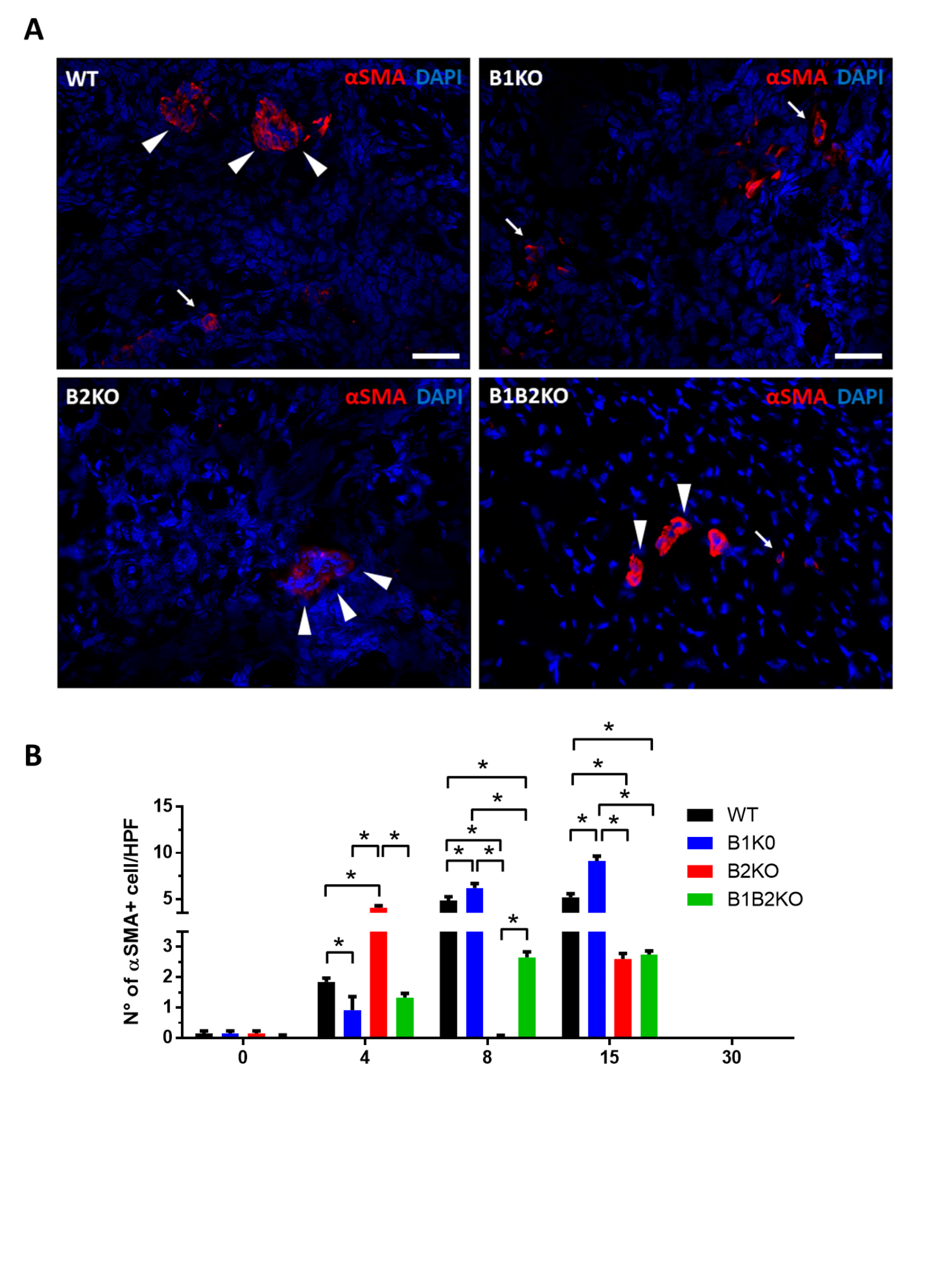


**Figure S5.** Change in Tibial anterior (TA) muscle weight normalized to the body weight. Data presented as mean ± SD.

**
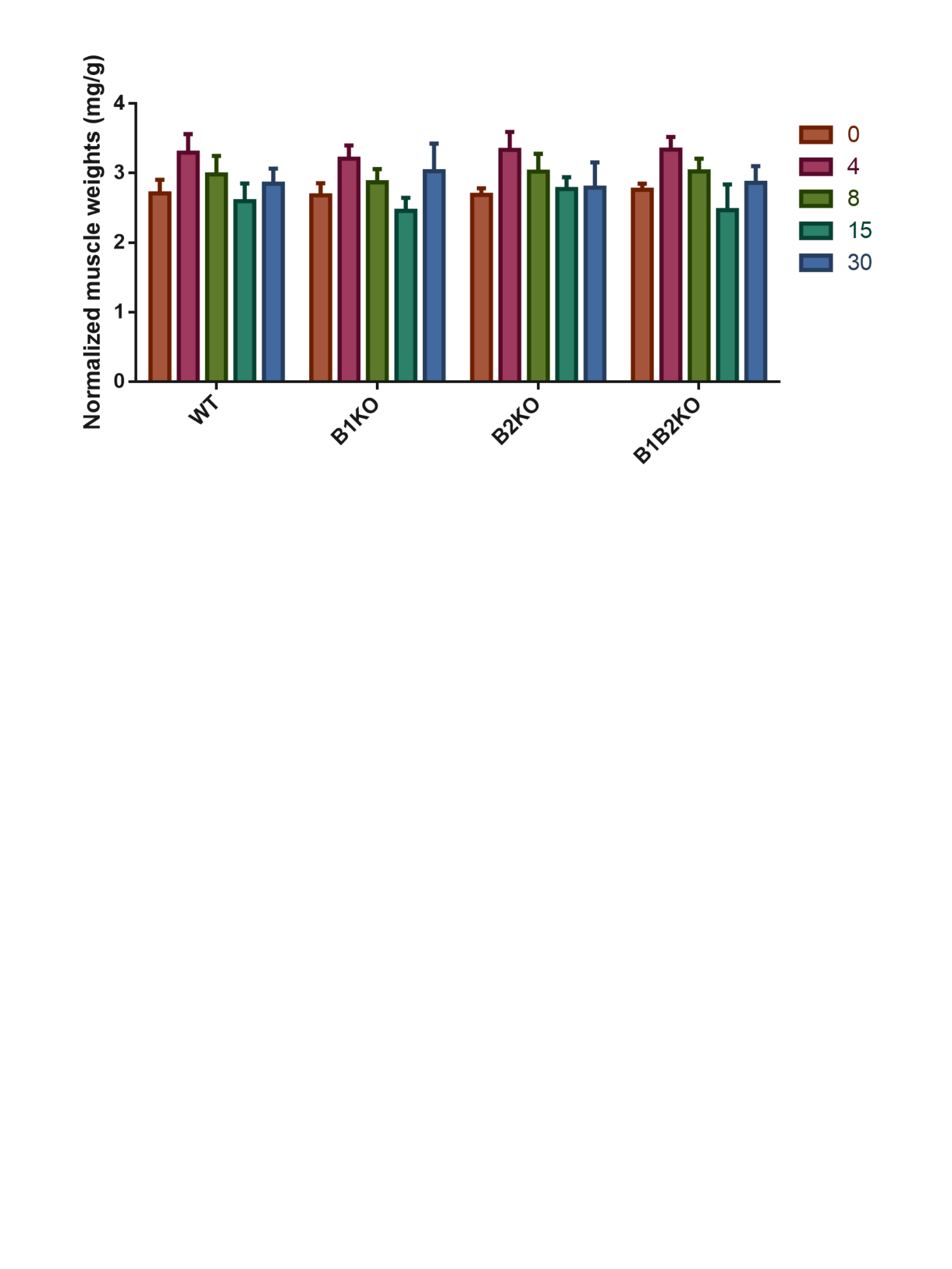
**

**Table S1.** Immunostaining protocols

|  | Procedure |
| --- | --- |
| **Fixation** | Acetone (10 min) |
| **Blocking** | 10% Normal Donkey Serum (NDS) (60 min; Invitrogen,  Carlsbad, CA) |
| **Permeabilization** | PBST (1x PBS+0.3% Triton) |
| **Step 1** | 1^st^ antibody (Dilution following the recommendations for each company in 1x PBS); overnight at 4°C. |
| **Step 2** | 2^nd^ antibody (1:300 in 1x PBS; 60 min; Molecular Probes) |
| **Step 3** | VECTASHIELD® Mounting Media with DAPI (4, 6‐Diamidino‐2‐  Phenylindole) (Vector Laboratories, Burlingame, CA, USA). |

**Table S2.** Antibodies used in immunofluorescence experiment.

| **Antibody** | **Reference** | **Host** | **Dilution** | **Company** |
| --- | --- | --- | --- | --- |
| *Primary antibodies* |  |  |  |  |
| anti-MyHC-emb | F1.652-c | Mouse | 1:5 | DSHB |
| anti-Col1A | Ab21286 | Rabbit | 1:100 | ABCAM |
| anti-α-SMA | Clone 1A4 | Mouse | 1:100 | Sigma-Aldrich |
| anti-Smad7 | [42-0400](https://www.thermofisher.com/order/genome-database/details/antibody/42-0400) | Rabbit | 1:200 | ThermoFischer |
| anti-p-Smad2/3 | #8828 | Rabbit | 1:200 | Cell Signaling |
| anti-MyoD1 | [5.8A](https://www.citeab.com/antibodies/search?cl=1&q=5.8A) | Rabbit | 1:50 | Dako |
| anti-CD69 | H1.2F3 | Hamster | 1:200 | BioLegend |
| anti-CD31 | ab56299 | Rat | 1:200 | ABCAM |
| anti-F4/80 | BM8 | Rabbit | 1:100 | BioLegend |
| *Secondary antibodies* |  |  |  |  |
| anti-rat Alexa Fluor® 488 | A-11006 | Goat | 1:300 | ThermoFischer |
| anti-rabbit Cy™3 | 711165152 | Donkey | 1:300 | Jackson ImmunoResearch Inc. |
| anti-hamster Alexa Fluor® 488 | A-21110 | Goat | 1:300 | ThermoFischer |

**Table S3.** Primers and probes for RT-qPCR.

| **Gene** | **Forward primer** | **Reverse primer** |
| --- | --- | --- |
| *Col1a1* | GACATGTTCAGCTTTGTGGACCTC | GGGACCCTTAGGCCATTGTGTA |
| *Tgfb1* | TGCGCTTGCAGAGATTAAAA | CTGCCGTACAACTCCAGTGA |
| *Mmp9* | ACGGACCCGAAGCGGACATT | TTGCCCAGCGACCACAACTC |
| *Il-6* | CTCTGGGAAATCGT GAAATG | AAGTGCATCATCGTTGTTCATACA |
| *Actb* | AGGGAAATCGTGCGTGACAT | GAACCGCTCGTTGCCAATAG |
| *Smad2* | Assay ID: Mm00487530_m1 | _ |
| *Smad7* | Assay ID: Mm00484742_m1 | _ |
| *Pax7* | Assay ID: Mm01354484_m1 | _ |
| *Myod1* | Assay ID: [Mm00440387_m1](https://www.thermofisher.com/taqman-gene-expression/product/Mm00440387_m1?CID=&ICID=&subtype=) | _ |
| *Vegfa* | Assay ID: Mm00437306_m1 | _ |
| *Ncl* | Assay ID: Mm01290600_g1 | _ |
| *Actb* | Assay ID: Mm02619580_g1 | _ |
| *Gapdh* | Assay ID: Mm99999915_g1 | _ |

**SI References**

[1] G. E. Truett, P. Heeger, R. L. Mynatt, A. A. Truett, J. A. Walker, and M. L. Warman, “Preparation of PCR-quality mouse genomic dna with hot sodium hydroxide and tris (HotSHOT),” *Biotechniques*, vol. 29, no. 1, pp. 52–54, 2000.

[2] C. Kasemkijwattana *et al.*, “Development of approaches to improve the healing following muscle contusion,” *Cell Transplant.*, vol. 7, no. 6, pp. 585–598, Nov. 1998.

[3] L. Martins *et al.*, “Skeletal muscle healing by M1-like macrophages produced by transient expression of exogenous GM-CSF,” *Stem Cell Res. Ther.*, vol. 11, no. 1, p. 473, Dec. 2020.

[4] W. Shen, Y. Li, Y. Tang, J. Cummins, and J. Huard, “NS-398, a cyclooxygenase-2-specific inhibitor, delays skeletal muscle healing by decreasing regeneration and promoting fibrosis,” *Am. J. Pathol.*, vol. 167, no. 4, pp. 1105–1117, 2005.

[5] W. Foster, Y. Li, A. Usas, G. Somogyi, and J. Huard, “Gamma interferon as an antifibrosis agent in skeletal muscle,” *J. Orthop. Res.*, vol. 21, no. 5, pp. 798–804, Sep. 2003.

[6] K. Fukushima, N. Badlani, A. Usas, F. Riano, F. H. Fu, and J. Huard, “The use of an antifibrosis agent to improve muscle recovery after laceration,” *Am. J. Sports Med.*, vol. 29, no. 4, pp. 394–402, 2001.

[7] J. M. G. S. C Kasemkijwattana, “Development of approaches to improve the healing following muscle contusion,” *Cell Transpl.*, vol. 7, pp. 585–598, 1998.

[8] F. F. Cunha, L. Martins, P. K. M. Martin, R. S. Stilhano, and S. W. Han, “A comparison of the reparative and angiogenic properties of mesenchymal stem cells derived from the bone marrow of BALB/c and C57/BL6 mice in a model of limb ischemia,” *Stem Cell Res. Ther.*, vol. 4, no. 4, p. 86, 2013.

[9] G. Li, H. Peng, K. Corsi, A. Usas, A. Olshanski, and J. Huard, “Differential Effect of BMP4 on NIH/3T3 and C2C12 Cells: Implications for Endochondral Bone Formation,” *J. Bone Miner. Res.*, vol. 20, no. 9, pp. 1611–1623, Sep. 2005.

[10] C. S. Day *et al.*, “Use of Muscle Cells to Mediate Gene Transfer to the Bone Defect,” *https://home.liebertpub.com/ten*, vol. 5, no. 2, pp. 119–125, Jan. 2007.

[11] J. Y. Lee *et al.*, “Enhancement of Bone Healing Based on *Ex Vivo* Gene Therapy Using Human Muscle-Derived Cells Expressing Bone Morphogenetic Protein 2,” *https://home.liebertpub.com/hum*, vol. 13, no. 10, pp. 1201–1211, Jul. 2004.

[12] L. Martins, P. K. M. Martin, and S. W. Han, “Angiogenic properties of mesenchymal stem cells in a mouse model of limb ischemia,” *Methods Mol. Biol.*, vol. 1213, pp. 147–169, 2014.

[13] T. A. Hornberger and R. P. Farrar, “Physiological Hypertrophy of the FHL Muscle Following 8 Weeks of Progressive Resistance Exercise in the Rat,” *Can. J. Appl. Physiol.*, vol. 29, no. 1, pp. 16–31, 2004.
